# CircSTX6 promotes pancreatic ductal adenocarcinoma progression by sponging miR-449b-5p and interacting with CUL2

**DOI:** 10.1186/s12943-022-01599-5

**Published:** 2022-06-01

**Authors:** Lingdong Meng, Yihan Zhang, Pengfei Wu, Danrui Li, Yichao Lu, Peng Shen, Taoyue Yang, Guodong Shi, Qun Chen, Hao Yuan, Wanli Ge, Yi Miao, Min Tu, Kuirong Jiang

**Affiliations:** 1 Pancreas Center, the First Affiliated Hospital of Nanjing Medical University, Nanjing, PR China; 2 Pancreas Institute, Nanjing Medical University, Nanjing, China; 3 Nanjing Medical University, Nanjing, China

**Keywords:** Pancreatic ductal adenocarcinoma, circSTX6, miR-449b-5p, HIF1A, MYH9

## Abstract

**Background:**

circular RNAs (circRNAs) have been reported to play crucial roles in the biology of different cancers. However, little is known about the function of circSTX6 (hsa_circ_0007905) in pancreatic ductal adenocarcinoma (PDAC).

**Methods:**

circSTX6, a circRNA containing exons 4, 5, 6 and 7 of the STX6 gene, was identified by RNA sequencing and detected by quantitative reverse transcription PCR (qRT–PCR). The biological function of circSTX6 was assessed in vitro and in vivo. The relationship between circSTX6 and miR-449b-5p was confirmed by biotin-coupled circRNA capture, fluorescence in situ hybridization (FISH) and luciferase reporter assays. The interaction of circSTX6 with Cullin 2 (CUL2) was verified by RNA–protein RNA pull-down, RNA immunoprecipitation (RIP) and western blotting assays.

**Results:**

circSTX6 was frequently upregulated in PDAC tissues, and circSTX6 overexpression promoted tumor proliferation and metastasis both in vitro and in vivo. Furthermore, circSTX6 expression was associated with tumor differentiation and N stage. Mechanistically, circSTX6 regulated the expression of non-muscle myosin heavy chain 9 (MYH9) by sponging miR-449b-5p. Moreover, circSTX6 was confirmed to participate in the ubiquitin-dependent degradation of hypoxia-inducible factor 1-alpha (HIF1A) by interacting with CUL2 and subsequently accelerating the transcription of MYH9.

**Conclusions:**

Our findings indicate that circSTX6 facilitates proliferation and metastasis of PDAC cells by regulating the expression of MYH9 through the circSTX6/miR-449b-5p axis and circSTX6/CUL2/HIF1A signaling pathway. Therefore, circSTX6 could serve as a potential therapeutic target for the treatment of PDAC.

**Supplementary information:**

The online version contains supplementary material available at 10.1186/s12943-022-01599-5.

## Introduction

Pancreatic cancer is one of the most malignant tumors in humans, with a 5-year survival rate of less than 9% [1–3]. A majority of patients with PDAC lose the opportunity to undergo radical resection due to local progression and distant metastasis [4]. Even for the fraction of patients who can undergo radical operation, only 37% of patients survive for 5 years due to early recurrence or distant metastasis [5, 6]. Thus, it is necessary to clarify the molecular mechanisms of PDAC.

Circular RNAs (circRNAs) are a new kind of noncoding RNA that are generated from a covalently closed continuous loop without a terminal 5’ cap and a 3’ polyadenylated tail [7]. CircRNAs exhibit higher stability than their linear counterparts because the loop structure is resistant to RNase R [8]. Recently, the biological functions of circRNAs in diverse cancers have been well explored, and it seems that the molecular mechanism of circRNAs is associated with their subcellular localization. CircRNAs in the nucleus could be involved in the regulation of gene transcription and the variable splicing of RNAs [9, 10], and circRNAs in the cytoplasm tend to serve as RNA sponges or interact with RNA binding proteins (RBPs) to participate in the posttranscriptional regulation of tumor-related genes [11]. However, the underlying mechanisms of circRNAs in PDAC remain elusive and require further study.

In the present study, we identified an oncogenic circRNA, circSTX6, through RNA sequencing and qRT–PCR. We demonstrated that circSTX6 was obviously upregulated in PDAC tissues and promoted proliferative and metastatic phenotypes in vivo and in vitro. Significantly, we found that circSTX6 could sponge miR-449b-5p to posttranscriptionally regulate the expression of MYH9. Moreover, we further revealed that circSTX6 suppressed the VHL-EloBC-CUL2-Rbx1 complex-dependent ubiquitination degradation of HIF1A by competitively combining with CUL2 and subsequently participating in the transcriptional regulation of MYH9. Collectively, our results reveal that the circSTX6/miR-449b-5p/MYH9 and circSTX6/CUL2/HIF1A/MYH9 axes play significant roles in PDAC progression. circSTX6 could serve as a potential biomarker of and therapeutic target for PDAC.

## Methods

### RNA-seq

For circRNAs sequencing, total RNA was extracted from five pairs of frozen PDAC tissues with different survival time using TRIzol Reagent (Life, CA, USA). RNA was then treated using a Ribo-off rRNA Depletion Kit (Vazyme, China) to remove ribosomal RNA before generating the RNA-seq library. Then, an illumina Novaseq^TM^6000 instrument (illumina, USA) was used to perform library sequencing. The FASTQ reads were aligned to the human reference genome (hg38/GRCh38). The counts of the remaining reads were normalized and mapped across an identified back-splice junction. The sequencing results of all differentially expressed circRNAs are shown in Table S1.

For miRNAs sequencing, adapters were ligated sequentially to the 3’ and 5’ ends of miRNAs in an unbiased reaction. miRNAs with adaptor were reversed-transcribed to create RNA-seq library. Then, library sequencing was performed on an illumina Novaseq^TM^6000 (illumina, USA) platform. Reads were mapped to the genome and miRNA were mapped to miRbase. The sequencing results of all differentially expressed miRNAs are shown in Table S2.

For mRNAs sequencing, mRNA with poly(A) was purified and interrupted and subsequently reversed-transcribed to create RNA-seq library. At last, we performed sequencing on an illumina Novaseq^TM^6000 (illumina, USA) system. Reads were mapped to the genome. The sequencing results of all differentially expressed mRNAs are shown in Table S3.

### Clinical samples

A total of 97 PDAC tissues and corresponding nontumor tissues were obtained from the First Affiliated Hospital of Nanjing Medical University between Jul. 2014 and Dec. 2018. None of these patients received chemotherapy or radiotherapy before surgery. All of the tumor samples and adjacent nontumor tissues were independently diagnosed by two pathologists. Overall survival was defined as the time between surgery and death. The hospital ethics committee approved this study, and informed consent was obtained from each patient prior to study participation.

### Cell culture

Three human PDAC cell lines (PANC-1, CFPAC-1, MIAPACA-2) and a normal human pancreatic ductal cell line (HPNE) were purchased from Shanghai Cell Bank (Shanghai, China). PDAC cell lines were cultured in Dulbecco’s modified medium (Wisent, Canada) supplemented with 10% fetal calf serum (Wisent, Canada). The HPNE cell line was grown in keratinocyte serum-free medium supplemented with epidermal growth factor and bovine pituitary extract according to the recommendations of the ATCC.

### RNA extraction and quantitative real-time PCR (qRT–PCR)

Total RNA was extracted from cells and tissue samples using TRIzol Reagent (Life, CA, USA) according to the manufacturer’s instructions. After spectrophotometric quantification, cDNA was synthesized from RNA according to the protocol of the commercial HiScript II Q RT SuperMix for qPCR kit (Vazyme, China). qRT–PCR was performed using the StepOne Plus RT PCR System (Thermo, USA) with SYBR Green (Vazyme, China). β-actin, GAPDH, 18 S rRNA and U6 were used as internal controls, and the levels of target genes were calculated based on the 2 ^−ΔΔCT^ method. The primers used in this study are listed in Table S4.

### RNA pull-down assay

A biotin-labeled circSTX6 probe (RiboBio, China) was incubated with streptavidin magnetic beads (RioBio, China) at room temperature for 2 h to generate probe-coated beads. Lysates from PANC-1 and CFPAC-1 cells were incubated with probe-coated beads at 4 °C overnight. Then, the beads were washed, and the pulled down miRNA was extracted and analyzed by qRT–PCR. The pulled down proteins were analyzed by mass spectrometry, silver staining and western blotting.

### RNA immunoprecipitation (RIP) assay

Anti-IgG, anti-AGO2 or anti-CUL2 antibodies were incubated with magnetic beads at room temperature for 30 min according to the instructions of the Magna RIP RNA-Binding Protein Immunoprecipitation Kit (Millipore, USA). Then, the antibody-bead complexes were incubated with cell lysates from PANC-1 and CFPAC-1 cells at 4 °C overnight. The bound RNAs were eluted and reverse transcribed to cDNA and subsequently detected by qRT–PCR.

### Fluorescence in situ hybridization (FISH) assay

Probes with 3′-Cy3 modification for circSTX6 and FAM-labeled probes specific to miR-449b-5p were synthesized (RioBio, China). Cells were first immobilized with 4% paraformaldehyde and then incubated with probes overnight at 37 °C. Nuclei were stained with DAPI and photographed by confocal microscopy (Carl Zeiss, Germany).

### Luciferase reporter assay

Wild-type and mutant MYH9 promoters, circSTX6, the 3′-untranslated region (3′-UTR) of MYH9, and collagen alpha-1 (XII) chain (COL12A1) fragments were constructed and subcloned into the luciferase reporter vector pRro-RB-Report. PDAC cells were co-transfected with different reporter gene plasmids and corresponding miRNA mimics or HIF1A plasmids. Then, the cells were harvested, and firefly luciferase (LUC) and Renilla luciferase (RLUC) activity was measured with the Dual-Luciferase Reporter System Kit (Promega, USA).

### Chromatin immunoprecipitation (CHIP) assay

The CHIP assay was constructed according to the instructions of the Magna CHIP™ A/G kit (Millipore, USA). Cells were fixed in 1% formaldehyde and then quenched in glycine, and the crosslinked chromatin was fragmented to 200–1000 bp by sonication. The lysates were incubated with the corresponding antibodies and Magna CHIP Protein G Magnetic beads at 4 °C overnight with gentle rocking. The CHIP complexes were eluted and analyzed by qRT–PCR. The data were calculated by the 2 ^−ΔΔCT^ method.

### Actinomycin D assay

PANC-1 or CFPAC-1 cells (1 × 10^5^) were seeded in a 6-well plate overnight and treated with 2 µg/ml actinomycin D (Sigma, USA) for 4, 8, 12, and 24 h. Cells were collected at the indicated time, and the expression of circSTX6 and STX6 mRNA was analyzed using qRT–PCR.

### RNase R treatment

RNase R (Lucigen, USA) was used for RNA digestion. Briefly, RNAs extracted from PANC-1 and CFPAC-1 cells were divided into two fractions. For RNase R digestion, 1 µg of RNA was treated with 2 U of RNase R. For the control, 1 µg of RNA was mixed with an equal volume of RNase-free water. Then, the expression of circSTX6 and linear STX6 mRNA was analyzed by qRT–PCR.

### Nuclear and cytoplasmic extraction

A PARIS™ kit (Thermo Fisher Scientific, USA) was used to isolate nuclear and cytoplasmic fractions. PANC-1 and CFPAC-1 cells were lysed in cell fraction buffer on ice and subsequently centrifuged at 500 g for 3 min at 4 °C, and the supernatant was collected as the cytoplasmic fraction. The pelleted nuclei were incubated with cell disruption buffer and used as the nuclear fraction. Then, the RNAs of these two fractions were extracted and detected by qRT–PCR.

### Plasmid construction, siRNA interference and lentiviral infection

Human circSTX6 cDNA was synthesized and cloned into the vector pLC5-ciR by Geneseed Biotech Co., Ltd. (Guangzhou, China). An empty plasmid served as the negative control. Two siRNA sequences were synthesized, and a scramble siRNA was synthesized as a negative control. Transfection was performed using Lipofectamine 3000 (Invitrogen, USA) according to the manufacturer’s instructions. Total proteins and RNA were collected 48 h after transfection.

Si-circSTX6-1 was subcloned into the vector pLV3ltr-Luc-Puro-U6-siRNA by Corues Biotechnology Corp., Ltd. (Nanjing, China) and verified by sequencing. The supernatant of the cultured 293T cells was collected to infect PANC-1 and CFPAC-1 cells. Stable cell lines were selected by 5 µg/ml puromycin (Sigma). The expression of circSTX6 was confirmed by qRT–PCR.

MiR-449b-5p mimics and inhibitors, plasmids and siRNAs of HIF1A and MYH9 were synthesized by Obio Technology (Shanghai) Corp., Ltd. and transfected as described above. All the sequences used in this study are presented in Table S4.

### Functional experiments

#### Cell count kit-8 (CCK-8) assay

Cells were seeded in 96-well plates at 2.0 × 10^3^ cells/well. Cell proliferation was assessed according to the protocol of the CCK-8 assay kit (Dojindo, Japan). Each day for the following 5 days, 10 µl of CCK-8 reagent was mixed with 90 µl of medium and added to each well. After incubation for 2 h at 37 °C away from light, the absorbance at 450 nm was measured in each well with a microplate reader.

### 5-Ethynyl-20- deoxyuridine (EdU) assay

 The EdU assay was performed according to the instructions of the EdU Proliferation Kit (RiboBio, China). Cells were seeded in 96-well plates, cultured for 24 h and then incubated with 50 mM EdU solution for 2 h in a cell incubator. After that, the cells were fixed in 4% paraformaldehyde, permeabilized with 0.3% Triton and subsequently stained with Apollo reaction buffer and Hoechst 33,342. The EdU-treated cells were imaged and counted under an Olympus FSX100 microscope (Olympus, Japan).

### Clone formation assay

Cells were plated in six-well plates (700 cells/well) and cultured in complete medium for 2 weeks. Subsequently, the cells were stained with 0.1% crystal violet (Beyotime, China) for 30 min and then washed with phosphate buffered saline (PBS). The colonies were counted if their diameter was greater than 1 mm.

### Wound healing assay

Cells were seeded in 6-well plates at a density of 7 × 10^5^ cells per well. When density reached approximately 100%, a straight scratch was made using a 200-µl pipette tip. The loose cells were removed by washing with PBS, and phase images were taken by inversion fluorescence microscopy (Olympus, Japan). ImageJ software was used to measure the relative wound areas.

### Transwell assay

Transwell assays were performed as previously described [12].

### Tissue microarrays and immunohistochemistry

Tissue microarrays and immunohistochemistry were performed as described in our previous study [13].

### Western blotting (WB)

Proteins were extracted from PDAC cells with RIPA buffer supplemented with PMSF and mixed with SDS loading buffer (Beyotime, China) before boiling for 5 min. Then, the proteins were separated by SDS–PAGE and transferred onto a PVDF membrane (Bio–Rad). After blocking the membranes with QuickBlock buffer (Beyotime, China) at room temperature for 20 min, the membranes were incubated with the appropriate antibody at 4 °C overnight. After washing with TBST buffer (Beyotime, China) three times, the membranes were incubated with the corresponding HRP-labeled secondary antibody (CST, USA) at room temperature for 2 h, after which they were washed again with TBST three times. Finally, the membranes were exposed and visualized with Chemiluminescence HRP Substrate (Millipore, USA). The antibodies used in this study are listed in Table S5.

### Animal studies

Four-week-old male nude mice (BALB/c) were purchased from the Animal Center of Nanjing Medical University. All the in vivo experiments were approved by the Committee on the Ethics of Animal Experiments of Nanjing Medical University. Six mice per group were used to construct subcutaneous tumor formation models. Stable cell lines transfected with circSTX6 KD virus and corresponding NC virus were constructed. 0.1 ml of cell suspensions containing 1 × 10^6^ stable cells (PANC-1-circSTX6 KD, PANC-1-circSTX6 KD NC, CFPAC-1-circSTX6 KD, CFPAC-1-circSTX6 KD NC) were injected subcutaneously into the axilla of the forelimb. When the tumor became macroscopic, the tumor size was measured every week for 35 days, and the formula (width^2^ × length)/2 was used to calculate the tumor volume.

For the tail vein tumor metastasis study, 0.1 ml of cell suspensions containing 1 × 10^6^ stable cells (PANC-1-circSTX6 KD, PANC-1-circSTX6 KD NC, CFPAC-1-circSTX6 KD, CFPAC-1-circSTX6 KD NC) were injected into the lateral tail veins of six mice of each group. The metastases were monitored using a Xenogen IVIS Spectrum Imaging System (PekinElmer, USA) at the indicated time (3 weeks, 4 weeks, 5 weeks) after injection. After that, the mice were euthanized, the lungs and livers were removed and fixed with 4% paraformaldehyde, each tissue was cut at the indicated three regions and the lung and liver metastatic nodules were validated using hematoxylin and eosin (HE)-stained sections by microscopy and presented as an average of three counts.

### Statistical analysis

Statistical analyses were performed using SPSS software (version 22.0), GraphPad Prism (version 6.01) and RStudio (version 4.0). The statistical significance of the differences between experimental groups was analyzed using Student’s t-tests. Quantitative data are presented as the means ± SD, and the correlations between the expression of circSTX6, miR-449b-5p, MYH9 and various clinicopathological or serological variables were analyzed by Х^2^ tests. The correlations between two variables were analyzed by linear regression. Survival distributions and overall survival rates were determined using the Kaplan–Meier method, and the significance of the differences between the survival rates was calculated by the log-rank test. Cox proportional hazards regression analysis was used to analyze the influential factors for prognosis. A difference was considered statistically significant if *p < 0.05, **p < 0.01, or ***p < 0.001.

## Results

### Identification and characteristics of circSTX6 in PDAC

**Fig. 1:**
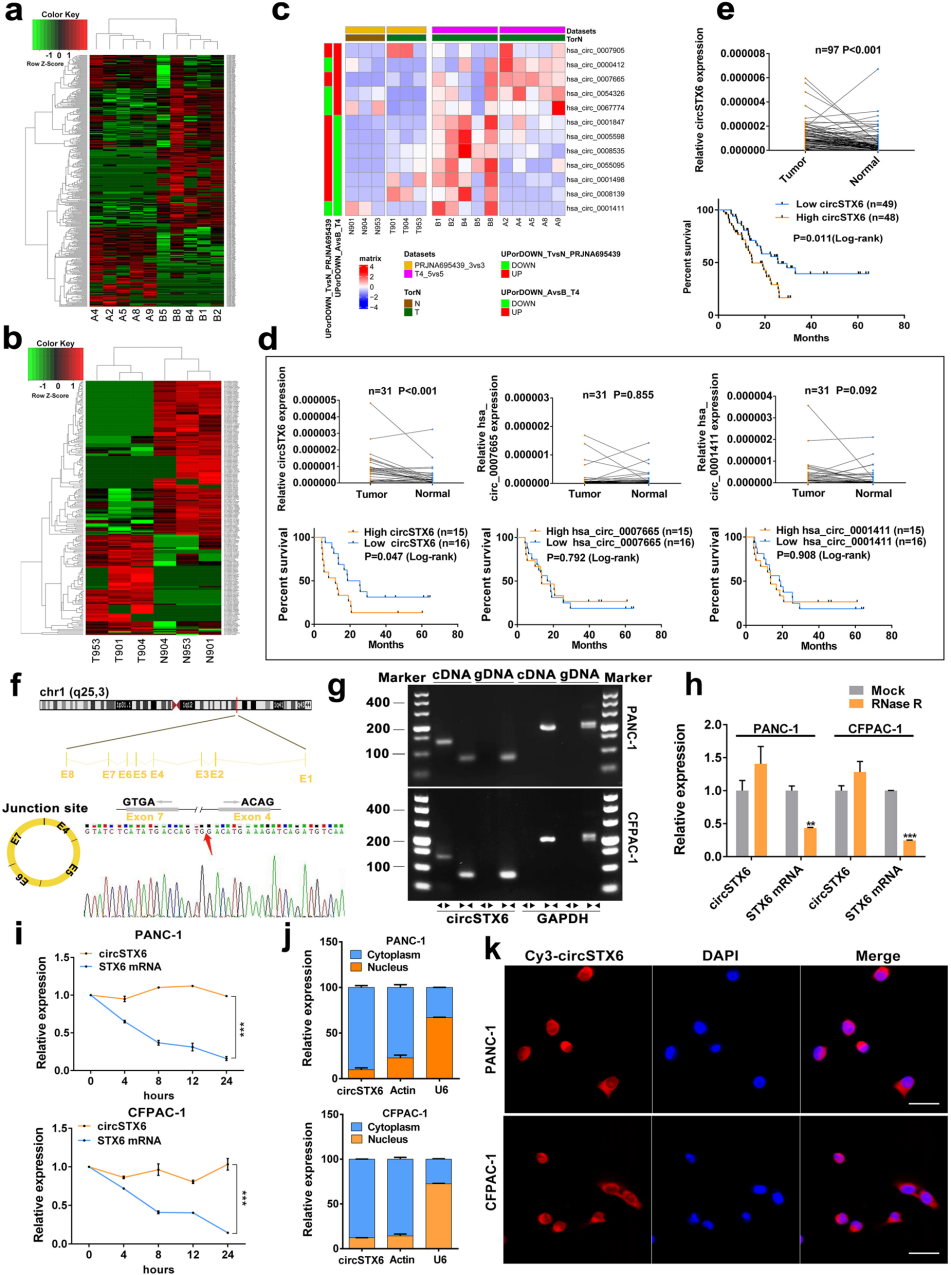
Identification and characterization of circSTX6 in PDAC cells and tissues. **a** Clustered heatmap of the differentially expressed circRNAs in PDAC tissues from five T4 PDAC patients with long survival time (Group B) and tissues from five T4 PDAC patients with short survival time (Group A). **b** Clustered heatmap of the differentially expressed circRNAs in three paired PDAC tissues (Group T) and matched noncancerous tissues (Group N). **c** Clustered heatmap of the intersection of two sequencing results. **d** Relative expression of three circRNA candidates (circSTX6, hsa_circ_0007665, hsa_circ_0001411) in 31 pairs of T4 PDAC tissues and corresponding noncancerous tissues. Kaplan–Meier analyses of the correlation between circRNA expression and overall survival were constructed. **e** Relative expression of circSTX6 in 97 pairs of PDAC tissues and corresponding noncancerous tissues. Kaplan–Meier analyses of the correlation between circSTX6 expression and overall survival were performed. **f** Schematic illustration of the genomic location and back splicing of circSTX6, with the splicing site validated by Sanger sequencing. **g** The existence of circSTX6 and STX6 were detected by qRT–PCR and agarose gel electrophoresis using divergent and convergent primers. **h** CircSTX6 and STX6 mRNA expression in PDAC cells were detected after RNase treatment compared to mock treatment. **i**. qRT–PCR analysis of circSTX6 and linear STX6 in PDAC cells treated with actinomycin D at the indicated time points. **j** Nuclear-cytoplasmic fractionation assay identified the localization of circSTX6. Actin and U6 were used as cytoplasmic and nuclear controls, respectively. **k** The subcellular location of circSTX6 in PDAC cells was investigated by FISH. Original magnification 400 ×. Scale bar = 50 μm. (Values are expressed as the means ± SDs; *P < 0.05, **P < 0.01 and ***P < 0.001)

To identify circRNAs that are crucial to PDAC progression, tissues from five T4 patients with long survival time and five corresponding T4 patients with short survival time were used for RNA-seq, and 407 circRNAs with p value less than 0.05 were identified. In the patients with longer survival time, 203 circRNAs were found to be upregulated, and 204 circRNAs were found to be downregulated (Fig. 1a). Combining our previous RNA-seq results of three PDAC and adjacent normal pancreas tissues (Fig. 1b) [14]. we obtained three prognosis-related and anomalously expressed circRNAs, hsa_circ_0007905 (named circSTX6), hsa_circ_0007665, and hsa_circ_0001411 (Fig. 1c). Next, we performed qRT–PCR to verify these three differentially expressed circRNAs in 31 pairs of T4 stage PDAC and corresponding nontumor tissues. We found that only circSTX6 was upregulated in PDAC (*p* < 0.001) and related to prognosis (Fig. 1d). The same results were obtained in a larger sample consisting of 97 patients (*p* < 0.001, Fig. 1e). Additionally, the expression level of circSTX6 was correlated with tumor differentiation and N stage (Tumor differentiation *p* = 0.008, N stage *p* = 0.019, Table 1), and circSTX6 was verified as a risk factor for overall patient survival in multivariable analysis (*p* < 0.001, Table 2).

CircSTX6 is generated from the STX6 gene located on chr1 (q25, 3). The circBase annotation for circSTX6 includes 4, 5, 6 and 7 exons. Sanger sequencing of the qRT–PCR products with divergent primers was used to verify its back-splicing (Fig. 1f). To further confirm the circular form of circSTX6, agarose gel electrophoresis analyses of the qRT–PCR products with divergent and convergent primers were performed, and the results showed that circSTX6 was amplified only by divergent primers in cDNA (Fig. 1g). We further confirmed that circSTX6 remained more stable than linear STX6 after treatment with RNase R and actinomycin D (Fig. 1h-i). Furthermore, the results of FISH and subcellular fractionation assays revealed that circSTX6 was mainly located in the cytoplasm of PDAC cells (Fig. 1j-k). The above results suggest that circSTX6 is overexpressed in PDAC tissues and mainly located in the cytoplasm of PDAC cells.

**Table 1:** Correlation between clinicopathological features of PDAC and expression of circSTX6, miR-449b-5p (*n* = 97)

Characteristics	circSTX6 expression			miR-449b-5p expression		
	Low expression (*n* = 49)	High expression (*n* = 48)	*P* value	Low expression (*n* = 49)	High expression (*n* = 48)	*P* value
Gender			0.941			0.617
Male	32	31		33	30	
Female	17	17		16	18	
Age(years)			0.667			0.162
< 60	12	10		14	8	
≥ 60	37	38		35	40	
Location			0.437			0.437
Head	30	33		30	33	
Body + tail	19	15		19	15	
Tumor differentiation			0.008**			0.584
G1 + G2	25	12		20	17	
G3	24	36		29	31	
TNM stage			0.264			0.019*
I + II	22	27		19	30	
III + IV	27	21		30	18	
T stage			0.352			0.01*
T1 + T2	25	29		21	33	
T3 + T4	24	19		28	15	
N stage			0.019*			0.752
N0	28	16		23	21	
N1 + N2	21	32		26	27	
Nerve invasion			0.102			0.765
No	8	2		6	4	
Yes	41	46		43	44	
Major vascular invasion			0.612			0.068
No	14	16		11	19	
Yes	35	32		38	29	
Serum CA19-9(U/ml)			0.424			0.812
< 37	9	6		8	7	
≥ 37	40	42		41	41	

*P < 0.05, **P < 0.01, ***P < 0.001. G1 stands for well differentiation, G2 stands for moderately differentiation, G3 stands for poor differentiation

**Table 2:** Univariate and multivariate analyses of overall survival in PDAC patients (*n* = 97)

Variables	Univariable analysis				Multivariable analysis		
	HR	95%CI	*P* value		HR	95%CI	*P* value
Gender (Female vs. Male)	0.838	0.470–1.494	0.549				
Age (≥ 60y vs. < 60y)	1.071	0.562–2.041	0.834				
Location (Body + tail vs. Head)	0.635	0.344–1.171	0.146				
Tumor_differentiation (G3 vs. G1 + G2)	1.015	0.578–1.781	0.96				
TNM stage (III + IV vs. I + II)	1.479	0.846–2.588	0.17				
T stage (T3 + T4 vs. T1 + T2)	1.882	1.086–3.262	0.024*		1.621	0.870–3.021	0.128
N stage (N1 + N2 vs. N0)	1.022	0.593–1.761	0.939				
Nerve invasion (Yes vs. No)	2.038	0.634–6.549	0.232				
Major vascular invasion (Yes vs. No)	1.907	0.957–3.801	**0.067**		1.363	0.633–2.936	0.428
Serum CA19_9 (≥ 37U/ml vs. < 37U/ml)	1.442	0.615–3.380	0.399				
CircSTX6 expression (High vs. Low)	2.025	1.162–3.528	0.013*		3.013	1.655–5.486	< 0.001***
miR-449b-5p expression (High vs. Low)	0.47	0.269–0.820	0.008**		0.426	0.232–0.780	0.006**

*HR* hazard ratio, *CI *confidence interval, *TNM *tumor node metastasis. Cox regression analysis, *p < 0.05, **p < 0.01, ***p < 0.001

### CircSTX6 promotes the proliferation, migration and invasion of PDAC cells in vitro

**Fig. 2:**
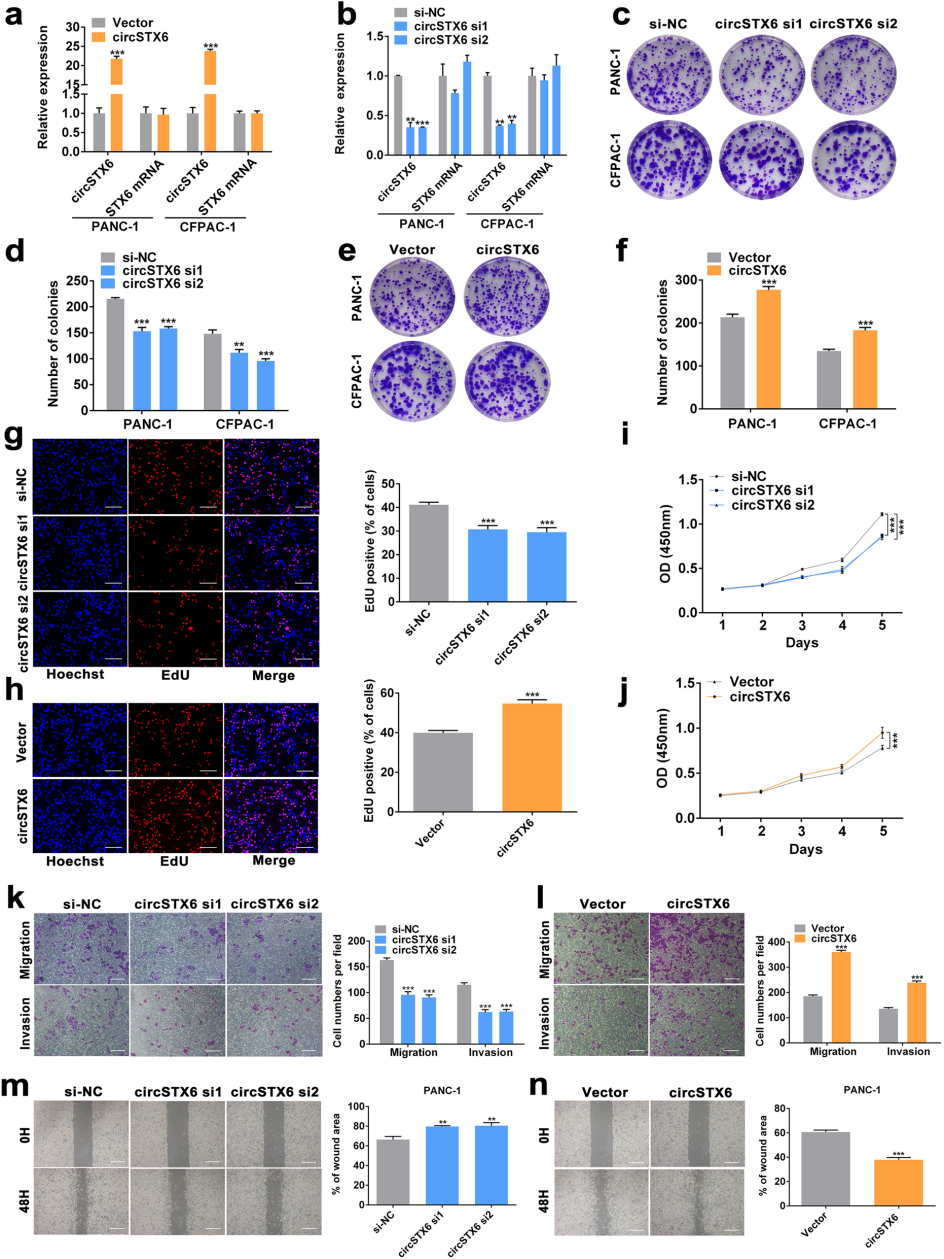
CircSTX6 promotes the proliferation, migration and invasion of PDAC cells in vitro. **a-b** qRT–PCR analysis of circSTX6 and linear STX6 expression in PANC-1 and CFPAC-1 cells transfected with circSTX6 siRNAs and circSTX6 plasmid. **c-f** Colony formation assays were performed in PDAC cells transfected with cirSTX6 siRNAs or plasmid. **g-h** EdU assays of PANC-1 cells were performed to evaluate cell proliferation. Original magnification 400 ×. Scale bar = 50 μm. **i-j** The growth curves of cells were evaluated by CCK-8 assays after knocking down and overexpressing circSTX6 in PANC-1 cells. **k-l** Transwell assays in circSTX6-knockdown and circSTX6-overexpressing PANC-1 cells. Original magnification 200 ×. Scale bar = 100 μm. **m-n** Wound healing assays were used to investigate the migratory ability of circSTX6-overexpressing and circSTX6-knockdown PANC-1 cells. Original magnification 200 ×. Scale bar = 100 μm. (Values are expressed as the means ± SDs; *P < 0.05, **P < 0.01 and ***P < 0.001)

First, we detected the expression of circSTX6 in HPNE and PDAC cell lines. We found that circSTX6 was highly expressed in PDAC cells, and PANC-1 and CFPAC-1 cell lines were used for further study (Fig. S1a). To assess the biological function of circSTX6 in PDAC cells, short interfering RNAs targeting the splice junction were synthesized to knockdown the expression of circSTX6, and the circSTX6 plasmid was constructed to overexpress circSTX6 without affecting the expression of STX6 mRNA. The efficiency of circSTX6 knockdown and overexpression was verified by qRT–PCR (Fig. 2a-b). Cell viability was further evaluated with CCK-8, EdU and clone formation assays, which indicated that circSTX6 knockdown suppressed proliferation and circSTX6 overexpression promoted proliferation (Fig. 2c-j and Fig. S1b-e). Moreover, the results of the wound healing assay and transwell assay revealed that the migratory and invasive abilities of PDAC cells were prominently decreased by circSTX6 knockdown and increased by circSTX6 overexpression (Fig. 2k-n and Fig. S1f-i). Taken together, these findings illustrate the oncogenic role of circSTX6 in PDAC cells.

### CircSTX6 promotes the tumorigenesis and metastasis of PDAC cells in vivo

**Fig. 3:**
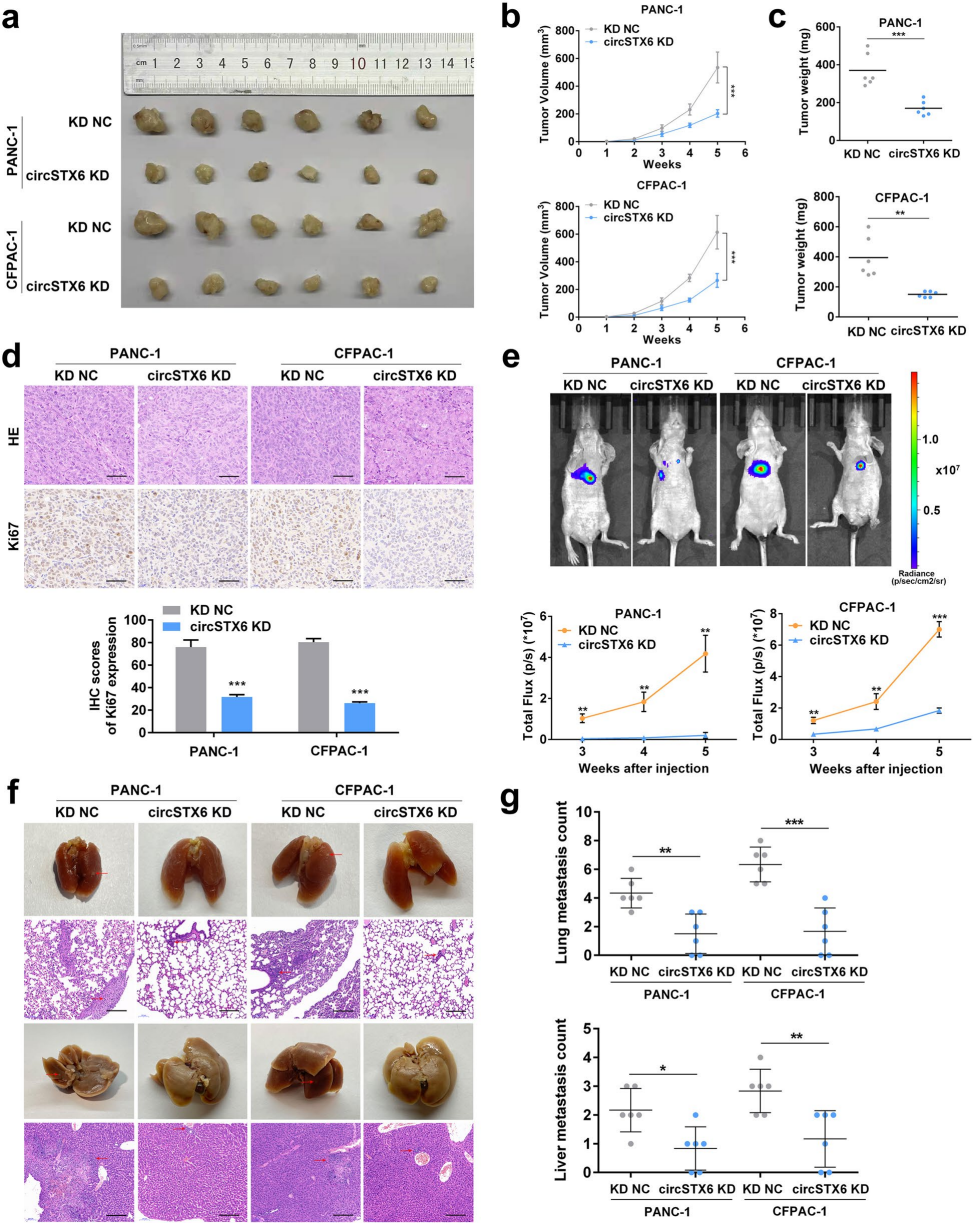
CircSTX6 facilitates the tumorigenesis and metastasis of PDAC cells in vivo. **a** Representative picture of subcutaneous xenograft tumors (*n* = 6 for each group). **b-c** Curves of tumor volumes and weights show negative effects of circSTX6 knockdown on the formation of subcutaneous xenograft tumors. **d** HE and Ki-67 IHC staining of xenograft tumors. Original magnification 400×. Scale bar = 50 μm. H-scores of the Ki67 staining. **e** Representative images and analysis of luminescence intensity in tail vein tumor metastasis mouse models (*n* = 6 for each group). **f** Representative images and HE staining of metastatic nodules in the lungs and livers of mice. Original magnification 100×. Scale bar = 200 μm. **g** The numbers of lung and liver metastatic nodules were measured. (Values are expressed as the means ± SDs; *P < 0.05, **P < 0.01 and ***P < 0.001)

To further evaluate the effect of circSTX6 on PDAC in vivo, stable PDAC cells transfected with LV-si-circSTX6 (circSTX6 KD) and the corresponding negative control (KD NC) were subcutaneously injected into BALB/cA nude mice. For subcutaneous tumor formation model studies, circSTX6 knockdown strongly inhibited tumor growth in both cell lines (Fig. 3a). Compared to the control group, the circSTX6 knockdown group showed significantly lower tumor volumes and weights (Fig. 3b-c). Additionally, a decreased Ki-67 level was observed in immunohistochemistry (IHC) staining in the circSTX6 knockdown group (Fig. 3d). In the study of tail vein tumor metastasis models, in vivo bioluminescence imaging demonstrated that the fluorescence intensity and proportion in the lungs and livers were significantly lower in the circSTX6 KD group than in the control group (Fig. 3e). Moreover, circSTX6 knockdown significantly decreased the number of metastatic nodules in the lungs and livers (Fig. 3f-g). The results above suggest that circSTX6 can promote the proliferation and metastasis of PDAC in vivo.

### CircSTX6 functions as a sponge of miR-449b-5p

**Fig. 4:**
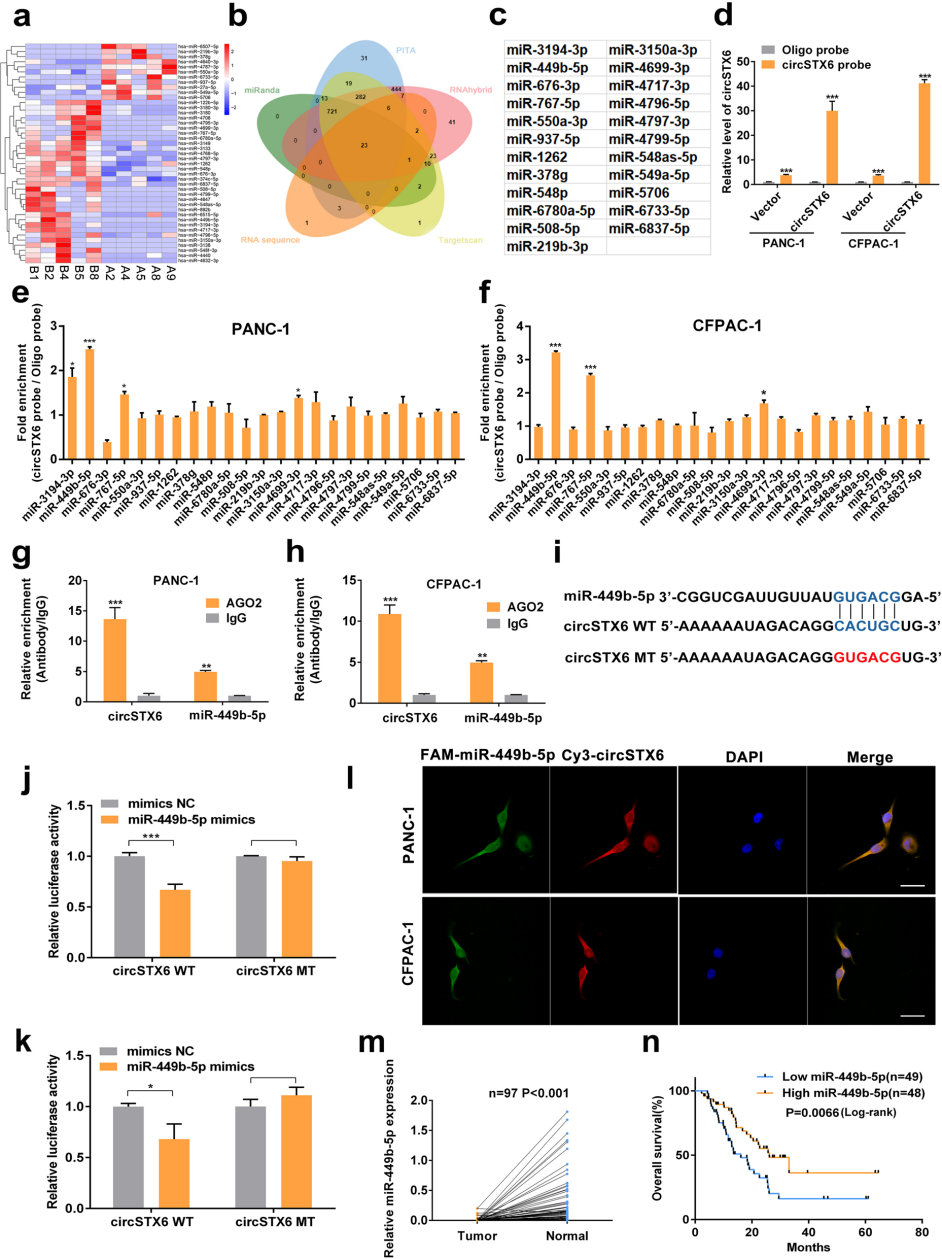
CircSTX6 serves as a sponge for miR-449b-5p. **a** Clustered heatmap of the differentially expressed miRNAs in PDAC tissues from five T4 PDAC patients with long survival time and tissues from five T4 PDAC patients with short survival time. **b** Venn diagram showing the overlap of the target miRNAs of circSTX6 predicted by miRanda, PITA, RNAhybrid, TargetScan and the results of RNA-seq. **c**. Table of target miRNAs selected for circSTX6. **d** The efficiency of the circSTX6 probe in PDAC cells was validated using qRT–PCR after the RNA pull-down assay. A random oligo probe served as a negative control. **e-f** The expression levels of 23 miRNA candidates were detected in the RNAs pulled down by circSTX6 and oligo probes. **g-h** Anti-AGO2 RIP was performed to detect circSTX6 and miR-449b-5p in PDAC cells. **i** A schematic of the wild-type (WT) and mutant (MUT) circSTX6 luciferase reporter vectors. **j-k** A luciferase reporter assay was used to confirm the interaction between circSTX6 and miR-449b-5p. **l** The colocalization of circSTX6 and miR-449b-5p in PDAC cells was detected using a FISH assay. The circSTX6 probe was labeled with Cy3 (red), the miR-449b-5p probe was labeled with FAM (green), and nuclei were stained with DAPI (blue). Original magnification 400 ×. Scale bar = 50 μm. **m** The expression of miR-449b-5p was detected in 97 PDAC tissues and corresponding noncancerous tissues. **n** Kaplan–Meier analysis of miR-449b-5p expression and overall survival in 97 patients with PDAC. The median expression value of miR-449b-5p was used as the cutoff (*n* = 49). Log-rank tests were used to determine the statistical significance. (Values are expressed as the means ± SDs; *P < 0.05, **P < 0.01 and ***P < 0.001)

The previous results indicated that circSTX6 was mainly expressed in the cytoplasm; therefore, we hypothesized that circSTX6 may serve as a miRNA sponge in PDAC. To identify the downstream miRNAs of circSTX6, we first performed a cross-analysis using four miRNA target prediction databases (miRanda, TargetScan, RNAhybrid and PITA) and the results of RNA-seq from five long-term survival T4 patients and five corresponding short-term survival T4 patients [15–18]. We identified 23 candidate miRNAs (miR-3194-3p, miR-449b-5p, miR-676-3p, miR-767-5p, miR-550a-3p, miR-937-5p, miR-1262, miR-378 g, miR-548p, miR-6780a-5p, miR-508-5p, miR-219b-3p, miR-3150a-3p, miR-4699-3p, miR-4717-3p, miR-4796-5p, miR-4797-3p, miR-4799-5p, miR-548as-5p, miR-549a-5p, miR-5706, miR-6733-5p, miR-6837-5p) that may bind to circSTX6 (Fig. 4a-c). A biotin-labeled circSTX6 probe was designed and constructed to pull down circSTX6 in two PDAC cell lines. The efficiency of the assay was examined with qRT–PCR, and the expression of circSTX6 was significantly upregulated in the circSTX6 transfectants, confirming that circSTX6 was successfully pulled down (Fig. 4d). Next, biotin-labeled circSTX6 was used to capture the potential binding miRNA in RNA pull-down assays, and miR-449b-5p was proven to be the most highly enriched miRNA in the sponge complexes for both PDAC cell lines (Fig. 4e-f). miRNA was reported to function as a RISC component by binding to Argonaute-2 (AGO2) [19, 20]. An RIP assay with anti-AGO2 was constructed, and the results showed that both circSTX6 and miR-449b-5p were pulled down by the anti-AGO2 antibody (Fig. 4g-h). Additionally, a dual-luciferase reporter assay was performed to verify the interaction between circSTX6 and miR-449b-5p. The results revealed that miR-449b-5p mimics observably attenuated the luciferase activity of wild-type circSTX6, and no difference was observed for circSTX6-mut (Fig. 4i-k). Moreover, the FISH assay indicated that circSTX6 and miR-449b-5p colocalized in the cytoplasm of PDAC cells (Fig. 4l and Fig. S1j-k). In general, these results demonstrate that circSTX6 physically interacts with miR-449b-5p in PDAC cells.

### CircSTX6 accelerates PDAC cell oncogenic effects by targeting miR-449b-5p

**Fig. 5:**
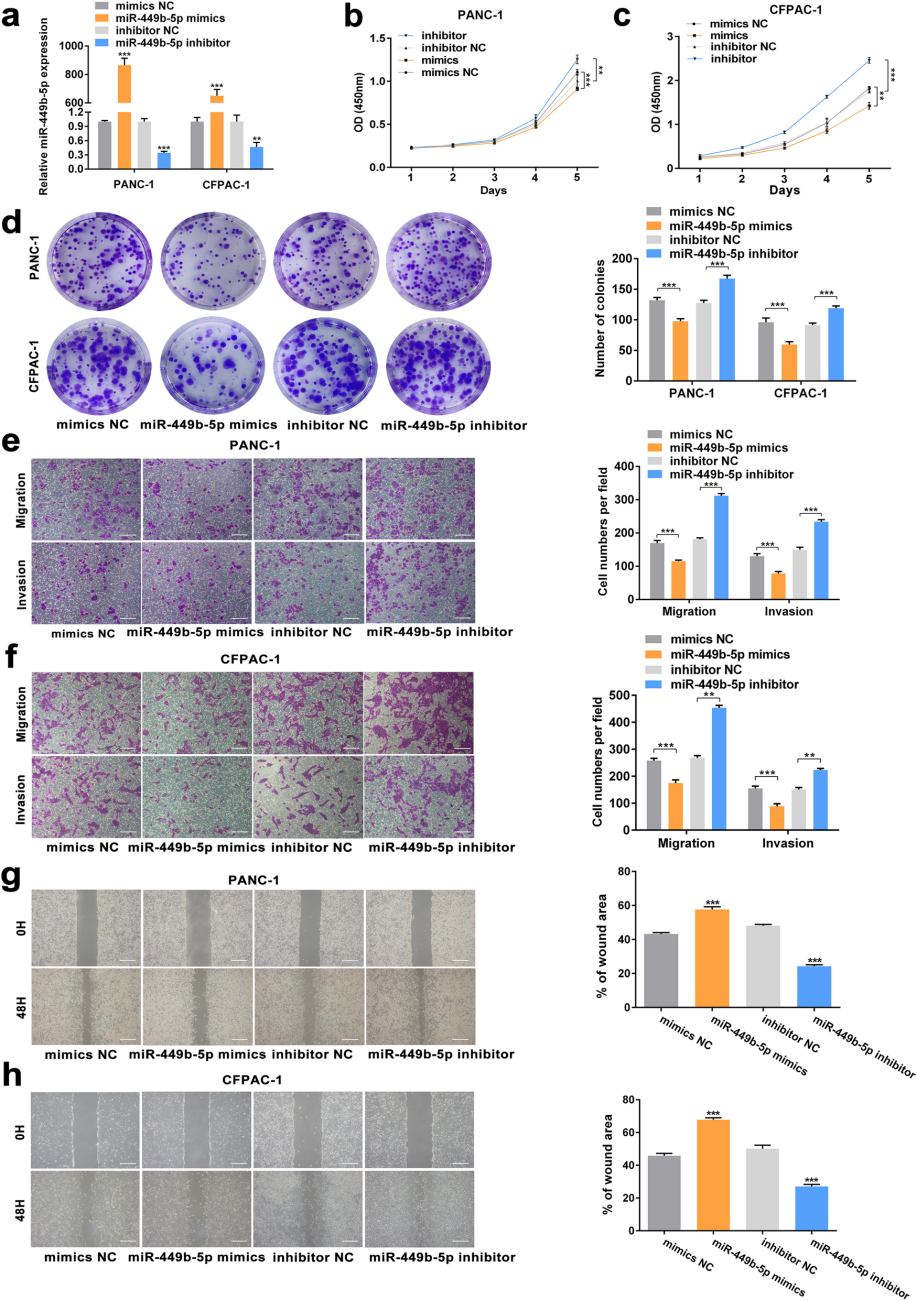
MiR-449b-5p suppresses the proliferation, migration and invasion of PDAC cells in vitro. **a** The construction of PDAC cells transfected with miR-449b-5p mimics and miR-449b-5p inhibitor. **b-d** CCK-8 and colony formation assays of PDAC cells were performed to evaluate cell proliferation. **e-h** Transwell and wound healing assays were used to investigate the migratory and invasive abilities of PDAC cells transfected with miR-449b-5p mimics and miR-449b-5p inhibitor. (**e**-**h**, original magnification 200 ×. Scale bar = 100 μm. Values are expressed as the means ± SDs; *P < 0.05, **P < 0.01 and ***P < 0.001)

We explored the expression of miR-449b-5p and found that miR-449b-5p was obviously downregulated in PDAC tissues compared to corresponding nontumor tissues (*p* < 0.001). Moreover, patients with higher expression levels of miR-449b-5p tended to have longer survival time (*p* = 0.0066, Fig. 4 m-n). Moreover, the expression level of miR-449b-5p was correlated with TNM stage and T stage (TNM stage *p* = 0.019, T stage *p* = 0.01, Table 1), and miR-449b-5p was verified as a protective factor for overall patient survival in multivariable analysis (*p* = 0.006, Table 2). Then, miR-449b-5p knockdown and overexpression cell lines were constructed (Fig. 5a). The results of CCK-8 and colony formation assays revealed that miR-449b-5p inhibitor significantly promoted and miR-449b-5p mimics significantly inhibited the proliferative abilities of PDAC cells (Fig. 5b-d). Moreover, the results of transwell and wound healing assays demonstrated that the migratory and invasive abilities of PDAC cells could be distinctly promoted with the transfection of miR-449b-5p inhibitor and suppressed by transfecting with miR-449b-5p mimics (Fig. 5e-h). To further investigate the roles of miR-449b-5p and circSTX6 in PDAC progression, rescue assays were performed. The results of transwell assays in PANC-1 and CFPAC-1 cells indicated that the promotive effects of circSTX6 overexpression on migratory and invasive abilities could be reversed by miR-449b-5p mimics and the suppressive effects induced by circSTX6 knockdown could be reversed by transfection with miR-449b-5p inhibitor (Fig. S2a-b). Similarly, the results of EdU and colony formation assays also revealed that miR-449b-5p mimics could reversed the promotive effects of circSTX6 overexpression on proliferative abilities of PDAC cells and miR-449b-5p inhibitor could reversed the proliferative abilities of PDAC cells suppressed by circSTX6 knockdown (Fig. S2c-e). Therefore, these data suggest that miR-449b-5p serves a significant function downstream of circSTX6.

### MYH9 is directly targeted by miR-449b-5p and indirectly regulated by circSTX6

**Fig. 6:**
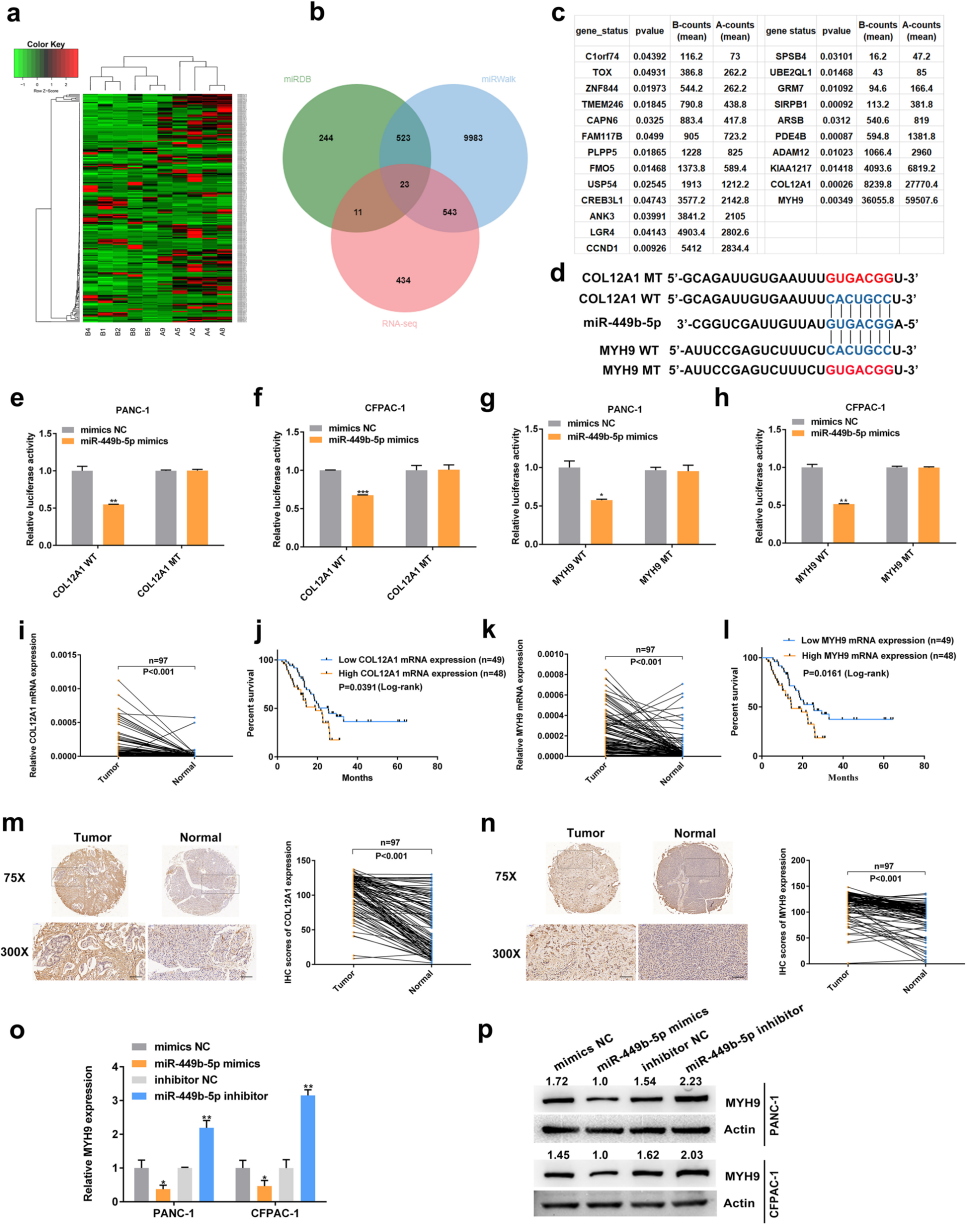
MYH9 is a direct target of miR-449b-5p. **a** Clustered heatmap of the differentially expressed mRNAs in PDAC tissues from five T4 PDAC patients with long survival time and tissues from five T4 PDAC patients with short survival time. **b** Venn diagram shows the overlap of the target mRNAs of miR-449b-5p predicted by miRDB, miWalk and the results of RNA-seq. **c** Table of target mRNAs selected for miR-449b-5p. **d** A schematic of the wild-type (WT) and mutant (MUT) COL12A1 or MYH9 luciferase reporter vectors. **e-f** A luciferase reporter assay was used to confirm the interaction between COL12A1 and miR-449b-5p. **g-h** A luciferase reporter assay was used to confirm the interaction between MYH9 and miR-449b-5p. **i-l** The expression levels of COL12A1 and MYH9 in PDAC tissues and matched noncancerous tissues were detected by qRT–PCR. Kaplan–Meier analyses were used to illuminate the correlation between the levels of COL12A1 and MYH9 and the survival time of PDAC patients. The median expression value of COL12A1 and MYH9 was used as the cutoff (n = 49). Log-rank tests were used to determine the statistical significance. **m-n** Representative COL12A1 and MYH9 IHC images of a tissue microarray containing 97 pairs of PDAC and corresponding noncancerous pancreatic tissues. Original magnification 75× and 300×. Scale bar = 100 μm. The protein levels of COL12A1 and MYH9 were significantly increased in PDAC tissues. **o-p** The expression levels of MYH9 mRNA (**o**) and protein (**p**) were significantly downregulated in PDAC cells transfected with miR-449b-5p mimics and upregulated in PDAC cells transfected with miR-449b-5p inhibitor. (Values are expressed as the means ± SDs; *P < 0.05, **P < 0.01 and ***P < 0.001)

To further explore the function of miR-449b-5p in PDAC, the results of RNA-seq combined with bioinformatic analysis using miRDB and miRWalk were used to analyze the downstream targets of miR-449b-5p [15, 21]. The data revealed 23 possible genes targeted by miR-449b-5p (Fig. 6a-c). After filtering out differentially expressed mRNAs with counts less than 5,000 and p value more than 0.01, COL12A1 and MYH9 were selected for the downstream analysis. Then, the wild-type and mutant dual-luciferase reporter plasmids harboring the wild-type or mutant COL12A1 3’-UTR or MYH9 3’-UTR were constructed (Fig. 6d). The results showed that transfection with miR-449b-5p mimics notably downregulated the luciferase activity in the plasmid with the WT but not the MUT 3’-UTR of COL12A1 and MYH9 (Fig. 6e-h). Mechanistically, COL12A1 and MYH9 shared the same sequence as circSTX6, which bound to the ‘seed’ region of miR-449b-5p. Moreover, the qRT–PCR results indicated that COL12A1 and MYH9 were highly upregulated in PDAC tissues (COL12A1 *p* < 0.001, MYH9 *p* < 0.001), higher expression levels of COL12A1 and MYH9 were correlated with poor prognosis in PDAC patients (COL12A1 p = 0.0391, MYH9 p = 0.0161, Fig. 6i-l). Similar results were observed in the IHC analysis (COL12A1 *p* < 0.001, MYH9 *p* < 0.001). However, the IHC results also demonstrated that COL12A1 was mostly located in fibroblasts but not in PDAC cells. We therefore selected MYH9 as the downstream target of miR-449b-5p (Fig. 6m-n).

**Fig. 7:**
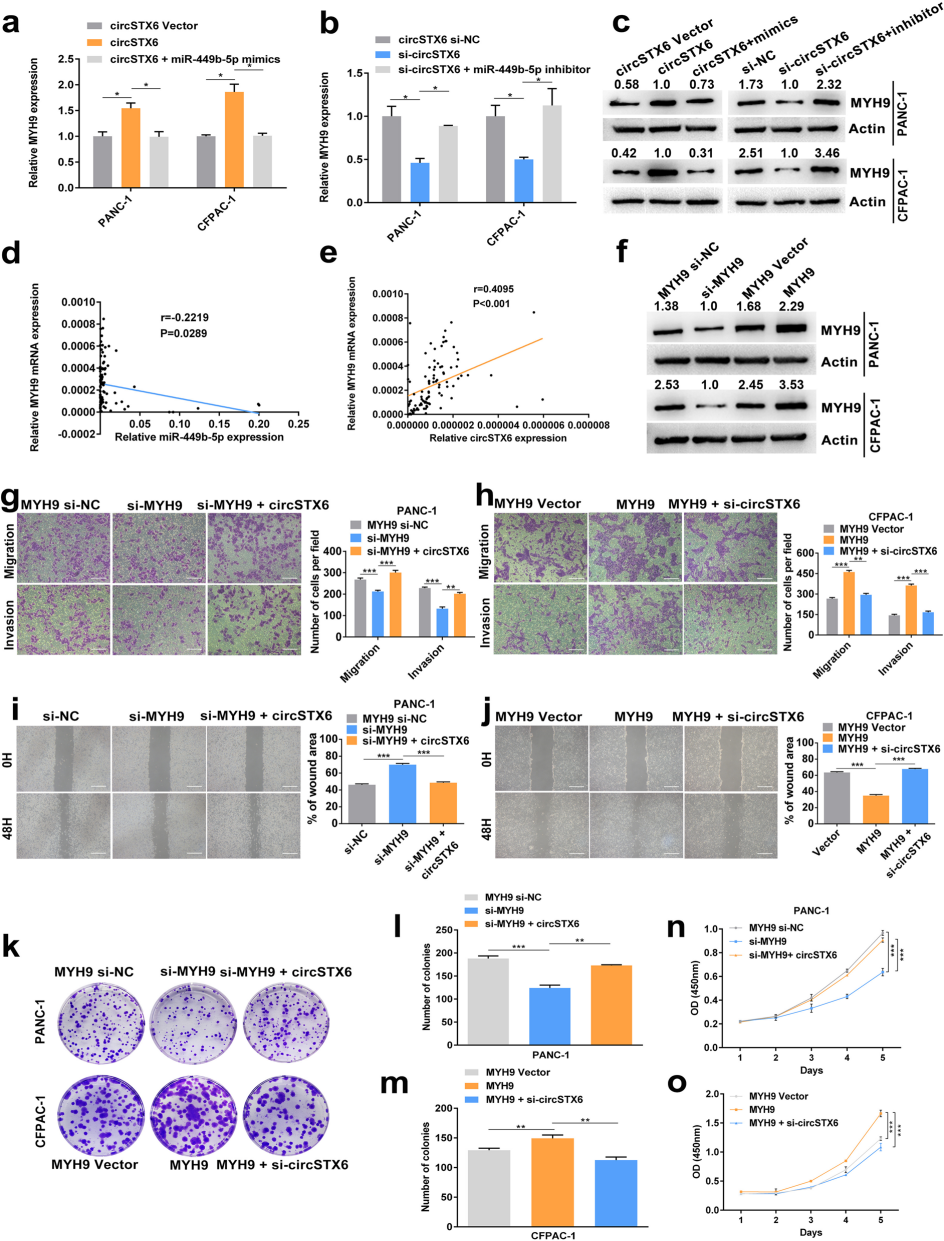
MYH9 is indirectly regulated by circSTX6. **a-c** The expression levels of MYH9 mRNA (**a-b**) and protein (**c**) were significantly upregulated in circSTX6-overexpressing and downregulated in circSTX6 knockdown PDAC cells, and these effects could be reversed by the transfection of miR-449b-5p mimics or inhibitor. **d** Correlation analysis showed a negative relationship between the levels of miR-449b-5p and MYH9. **e** Correlation analysis showed a positive correlation between the levels of circSTX6 and MYH9. **f** MYH9 knockdown and overexpression cell lines were constructed. **g-j** MYH9 knockdown suppressed and MYH9 overexpression promoted the migratory and invasive abilities of PDAC cells, and these effects could be rescued by the transfection of circSTX6 plasmid or siRNA. Original magnification 200 ×. Scale bar = 100 μm. **k-o** The results of colony formation and CCK-8 assays revealed that MYH9 knockdown suppressed and MYH9 overexpression promoted proliferative abilities of PDAC cells, and these effects could be reversed by the transfection of circSTX6 plasmid or siRNA. (Values are expressed as the means ± SDs; *P < 0.05, **P < 0.01 and ***P < 0.001)

The results of qRT–PCR and western blotting identified that MYH9 was negatively regulated by miR-449b-5p (Fig. 6o-p). Moreover, the mRNA and protein levels of MYH9 were positively regulated by circSTX6, which could be fully rescued by miR-449b-5p mimics or inhibitor (Fig. 7a-c). The qRT–PCR results of PDAC tissues indicated that the expression level of MYH9 in PDAC tissues was positively correlated with circSTX6 levels and negatively correlated with miR-449b-5p levels (circSTX6 *p* < 0.001, miR-449b-5p *p* = 0.0289, Fig. 7d-e). Then, MYH9 overexpression and knockdown cell lines were constructed (Fig. 7f). The results of transwell, wound healing, colony information and CCK-8 assays demonstrated the oncogenic effects of MYH9 in PDAC, and these effects could be rescued by circSTX6 overexpression or knockdown (Fig. 7 g-o). In summary, we confirm that circSTX6 upregulates the expression of MYH9 by sponging miR-449b-5p.

### CircSTX6 physically interacts with CUL2 in PDAC cells

**Fig. 8:**
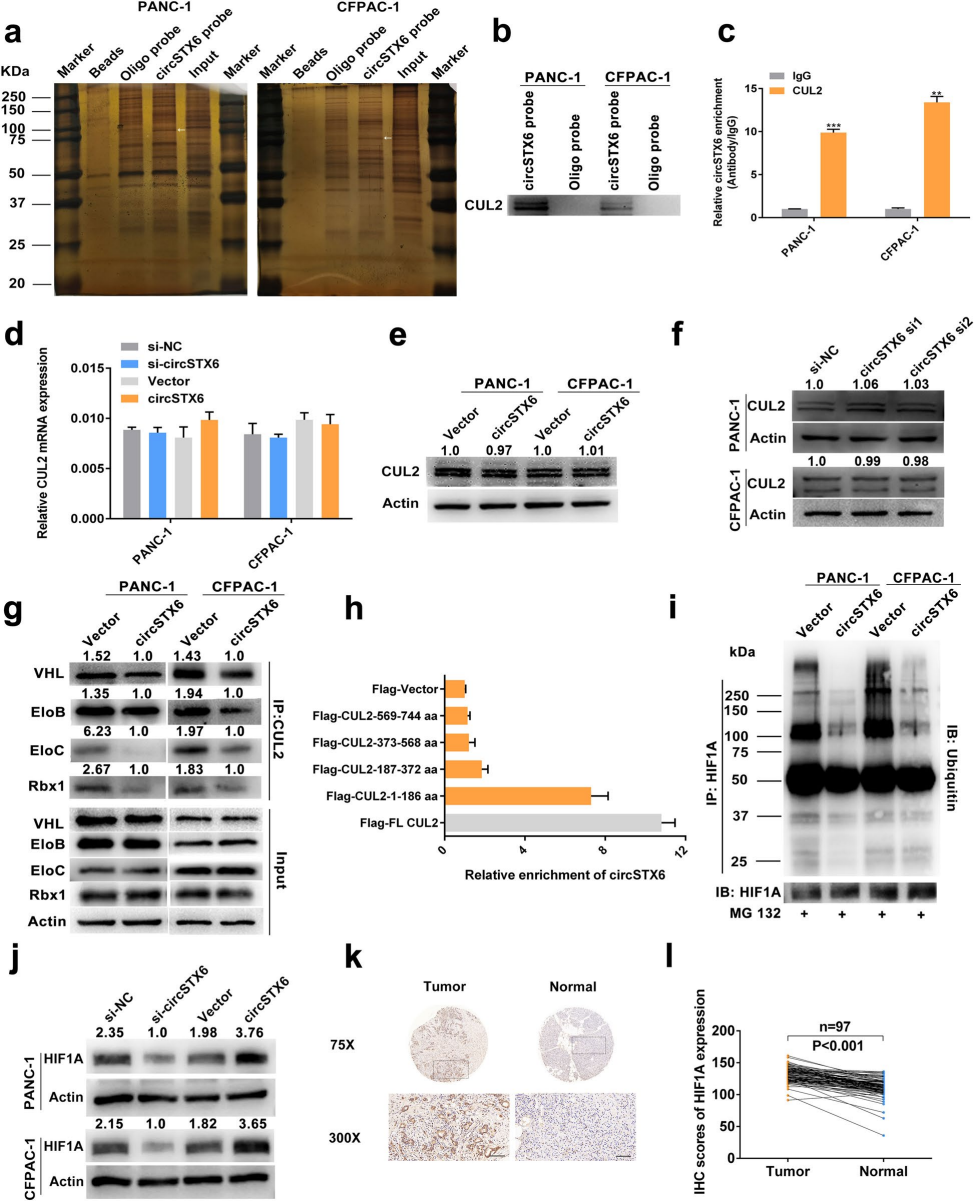
CircSTX6 physically interacts with CUL2 and participates in the regulation of HIF1A. **a** CircSTX6 and oligo probes were incubated with proteins extracted from PDAC cells for RNA pull-down assays. Proteins pulled down were used for silver staining, and a specific band appeared between 75 and 100 kDa (arrow). **b** Levels of CUL2 protein were detected in the proteins pulled down by circSTX6 and oligo probes. **c** RIP assays were performed using antibodies against CUL2 and IgG. **d-f** Levels of CUL2 mRNA and protein in circSTX6-overexpressing and circSTX6-knockdown PDAC cells were quantified by qRT–PCR and western blotting. β-actin served as the internal control. **g** The levels of interacting proteins immunoprecipitated by CUL2 were detected in circSTX6-overexpressing PDAC cells. **h** CUL2 was truncated (1–186 aa, 187–372 aa, 373–568 aa, and 569–744 aa) to identify the specific fragment of CUL2 that bound to circSTX6. RIP assays were performed to detect the enrichment of circSTX6 in cells transfected with full-length and truncated flag-tagged constructs. **i** HIF1A was first immunoprecipitated, and the ubiquitination level of HIF1A was detected in PDAC cells transfected with the circSTX6 plasmid. **j** The expression level of HIF1A was detected in circSTX6-overexpressing or circSTX6-knockdown PDAC cells. **k** Representative HIF1A IHC images of a tissue microarray containing 97 pairs of PDAC and corresponding noncancerous pancreatic tissues. Original magnification 75× and 300×. Scale bar = 100 μm. **l** The protein level of HIF1A was significantly increased in PDAC tissues. (Values are expressed as the means ± SDs; *P < 0.05, **P < 0.01 and ***P < 0.001)

CircRNAs have been reported to interact with RNA-binding proteins to participate in the development of different cancers. Therefore, we carried out an RNA–protein RNA pull-down assay to identify the interacting proteins of circSTX6. Proteins pulled down were sent for mass spectrometry and silver staining assays (Fig. 8a). 14 cytoplasm-located proteins, which were only detected in the circSTX6 probe group, were identified in the mass spectrometry analysis (Table S6). Moreover, a specific band was observed between 75 and 100 kDa, this specific band was cut, detected with mass spectrometry and subsequently identified as CUL2. Combining the results above, we hypothesized that CUL2 interacts with circSTX6. Western blotting of the proteins that were pulled down confirmed CUL2 as the direct binding protein of circSTX6 (Fig. 8b). Consistently, the results of RIP assays demonstrated that circSTX6 was markedly enriched in the precipitate obtained with the anti-CUL2 antibody, as determined by qRT–PCR (Fig. 8c).

**Fig. 9:**
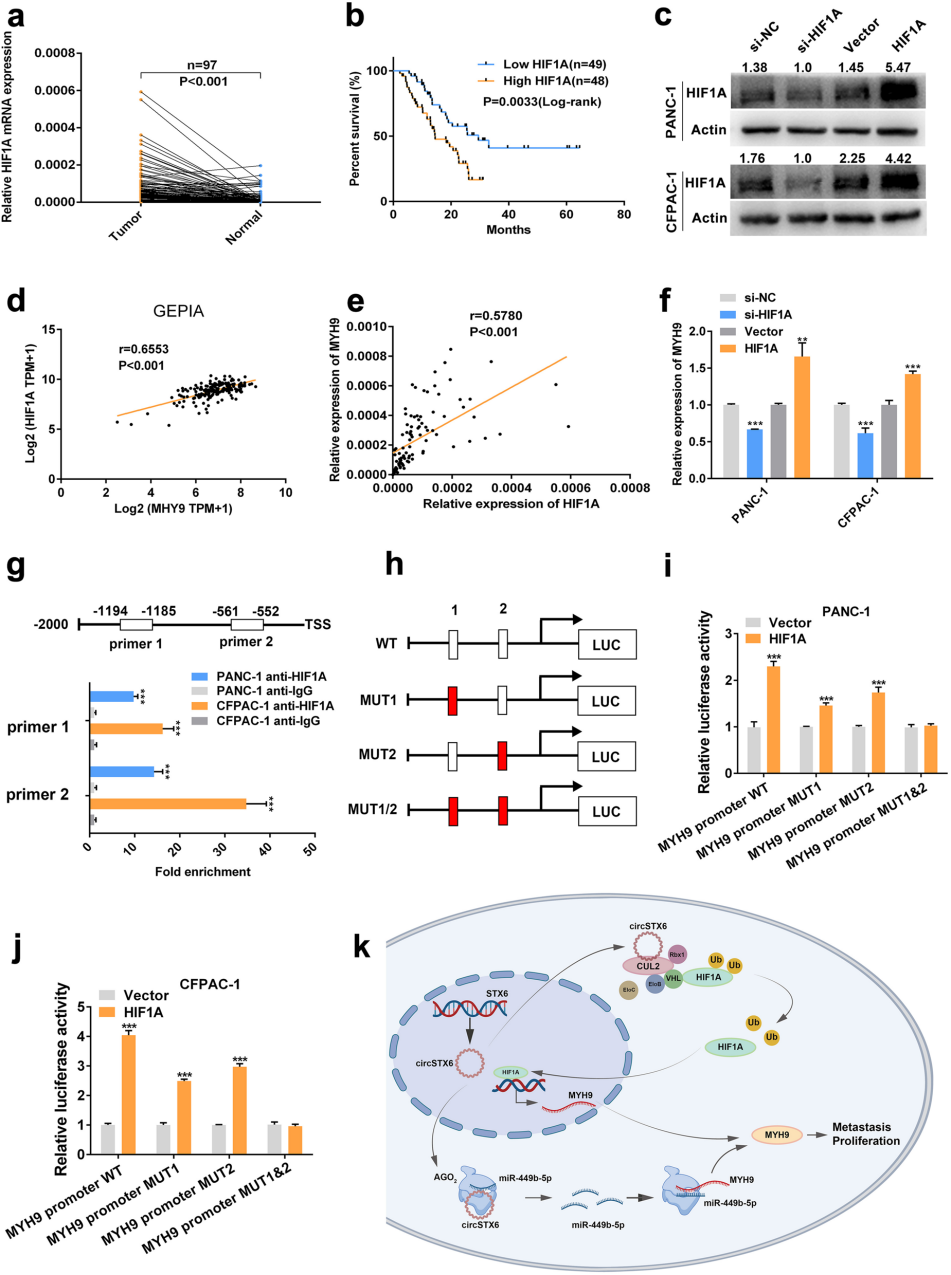
HIF1A promotes MYH9 transcription. **a-b**. The expression level of HIF1A in PDAC tissues and matched noncancerous tissues was detected by qRT–PCR. Kaplan–Meier analysis was used to illuminate the correlation between the level of HIF1A and the survival time of PDAC patients. The median expression value of HIF1A was used as the cutoff (*n* = 49). Log-rank tests were used to determine the statistical significance. **c**. HIF1A knockdown and overexpression cell lines were constructed. **d**. GEPIA analysis showed a positive relationship between MYH9 and HIF1A in PDAC tissues. **e**. Correlation analysis showed a positive relationship between MYH9 and HIF1A in 97 PDAC tissues. 18 S served as the internal control. **f**. MYH9 mRNA expression was significantly downregulated in HIF1A-knockdown PDAC cells and upregulated in HIF1A-overexpressing PDAC cells. **g**. CHIP assays revealed that HIF1A could bind to two fragments (-1194 – -1185, -561 – -552) of the MYH9 promoter. **h-j**. Luciferase reporter assays were used to confirm the interaction between HIF1A and two fragments (-1194 – -1185, -561 – -552) of the MYH9 promoter. **k**. Schematic diagram illustrating the mechanism by which circSTX6 promotes PDAC proliferation and metastasis through posttranscriptional and transcriptional regulation of MYH9 by sponging miR-449b-5p and inhibiting VHL-EloBC-CUL2-Rbx1 complex-dependent ubiquitination of HIF1A, respectively. (Values are expressed as the means ± SDs; *P < 0.05, **P < 0.01 and ***P < 0.001)

The results of qRT–PCR and western blotting revealed that circSTX6 exerted no effect on the mRNA and protein levels of CUL2 (Fig. 8d-f). With a literature review, we found that CUL2 was mainly involved in the ubiquitin-dependent degradation of HIF1A through the formation of the VHL-EloBC-CUL2-Rbx1 complex [22–24]. Therefore, we hypothesized that circSTX6 may participate in the formation of the VHL-EloBC-CUL2-Rbx1 complex by interacting with CUL2 and is subsequently involved in the regulation of HIF1A. The results of the co-IP assay demonstrated that the levels of VHL, EloB, EloC and Rbx1 immunoprecipitated by CUL2 were downregulated in the circSTX6 group compared to the vector group (Fig. 8g). Further RIP assays using full-length and truncated CUL2 showed that the 1-186 aa fragment of CUL2, which is responsible for the interaction of CUL2 with EloC, was mainly responsible for its interaction with circSTX6 (Fig. 8h). Moreover, our results demonstrated that cirSTX6 could suppress the ubiquitin-dependent degradation of HIF1A in PDAC cells (Fig. 8i). The western blotting results revealed that the level of HIF1A was obviously upregulated in cells overexpressing circSTX6 and downregulated in cells transfected with circSTX6 siRNAs (Fig. 8j). Moreover, we found that the protein and mRNA levels of HIF1A were severely upregulated in PDAC tissues and that patients with higher HIF1A expression tended to have poorer prognosis (Figs. 8k-l and 9a-b). The results of transwell and wound healing assays revealed that HIF1A overexpression promoted and HIF1A knockdown suppressed the migratory and invasive abilities of PDAC cells, and these effects could be reversed by transfection with circSTX6 plasmid or siRNAs (Fig. 9c and Fig. S3a-d). Consistently, the same results could be observed in clone formation and CCK-8 assays (Fig. S3e-i). Overall, we identify that circSTX6 suppresses the ubiquitin-dependent degradation of HIF1A by physically interacting with CUL2.

### HIF1A binds to the promoter region of MYH9

CircSTX6 has been confirmed to regulate the expression level of MYH9 by sponging miR-449b-5p, and we further proved that circSTX6 was involved in the ubiquitin-dependent degradation of HIF1A by physically interacting with CUL2. Therefore, we hypothesized that HIF1A participated in the regulation of MYH9. The data from GEPIA and the qRT–PCR results indicated that the expression of HIF1A was positively correlated with that of MYH9 (GEPIA *p* < 0.001, our center *p* < 0.001, Fig. 9d-e). Moreover, MYH9 was upregulated in cells overexpressing HIF1A and downregulated in cells transfected with HIF1A siRNAs (Fig. 9f). Thus, we hypothesized that HIF1A promoted the expression of MYH9 at the transcriptional level. The results of JASPAR analysis revealed that HIF1A may bind to the promoter region (-1194 – -1185, -561 – -552) of MYH9, and the subsequent results of CHIP demonstrated that these two fragments were significantly upregulated in the anti-HIF1A group compared to the IgG group (Fig. 9g). Furthermore, the results of luciferase reporter assays also showed that HIF1A could bind to these two regions of MYH9 (Fig. 9 h-j). In all, these results revealed that HIF1A can promote the transcription of MYH9 by binding to the promoter of MYH9.

## Discussion

Recently, a growing number of studies have confirmed the dysregulation of circRNAs, a kind of novel noncoding RNA, in a variety of cancers and clarified the involvement of circRNAs in the multiple modulation of cellular functions. In this study, for the first time, we identified a significantly upregulated and prognosis-related circRNA, circSTX6, in PDAC tissues. The correlative analyses between the clinical pathological or serological characteristics and circSTX6 expression showed that circSTX6 was statistically correlated with tumor differentiation and N stage. Moreover, multivariable analysis verified circSTX6 as a risk factor for overall patient survival. Loss- and gain-of-function experimental results revealed that circSTX6 promoted the proliferative, migratory and invasive abilities of PDAC cells in vitro and in vivo, indicating the tumorigenic role of circSTX6 in PDAC and its potential to predict the prognosis of PDAC patients.

Accumulating reported evidence indicates that circRNAs in the cytoplasm can act as miRNA sponges to induce the dysregulated function of miRNAs and their target genes. Guo. et al. reported that circKDM4B acts as a miR-675 sponge to suppress breast cancer progression [25]. Chen. et al. confirmed that circ101555 functions as a competing endogenous RNA of miR-597-5p to upregulate CDK6 and RPA3 expression in colorectal cancer [26]. In addition, Xie. et al. found that circular RNA BCRC-3 sponges miR-182-5p to promote the activity of p27 in bladder cancer [27]. In the present study, through bioinformatic analyses and a series of validation experiments, such as RNA pull-down, anti-AGO2 RNA immunoprecipitation, dual-luciferase and FISH, we verified the combination of circSTX6 and miR-449b-5p. The expression level of miR-449b-5p showed a positive correlation with TNM stage and T stage, and multivariable analysis revealed that miR-449b-5p was a protective factor for overall patient survival. Furthermore, the results of a rescue experiment showed that the promotion induced by circSTX6 overexpression and suppression induced by circSTX6 knockdown on the proliferative, migratory and invasive abilities of PDAC cells could be reversed by treatment with miR-449b-5p mimics or inhibitor, respectively. Collectively, our results reveal that circSTX6 can function as a miRNA sponge to participate in the proliferation and metastasis of PDAC.

Recent studies have verified that miR-449b-5p plays critical roles in modulating tumor development and progression. Guo. et al. reported that LINC01106 positively modulates Gli4 by sponging miR-449b-5p to drive colorectal cancer growth and stemness [28]. Additionally, miR-449b-5p was confirmed to inhibit the proliferation of glioblastoma by inactivating the WNT2B/Wnt/β-catenin signaling pathway [29]. To further elucidate the molecular mechanism of miR-449b-5p, we conducted bioinformatic analyses and selected COL12A1 and MYH9 as possible targets of miR-449b-5p. Through further dual-luciferase reporter, qRT–PCR and IHC staining analyses, we ultimately identified MYH9 as the downstream target of miR-449b-5p.

MYH9, also known as myosin or non-muscle myosin heavy chain 9, is a 226 kDa subunit of class II conventional myosin that has been reported to play dual functions in cancers [30, 31]. Studies have shown that higher expression of MYH9 is associated with a good prognosis in patients with head and neck squamous cell carcinomas [32]; however, a large amount of research has also revealed that MYH9 is involved in the biological process of solid tumors of the digestive tract, including gastric cancer, colorectal cancer and pancreatic cancer [33–35]. MYH9 was significantly upregulated in PDAC tissues. Moreover, the expression of MYH9 was positively correlated with that of circSTX6 or negatively correlated with that of miR-449b-5p. No significant difference was observed between the level of MYH9 and clinical pathological or serological characteristics due to the insufficient sample size. With an increase in the sample size, the prognostic effects of MYH9 may appear. These results demonstrated that circSTX6 participates in the posttranscriptional regulation of MYH9 by sponging miR-449b-5p.

CircRNAs have also been reported to regulate the tumor process by interacting with RNA binding proteins (RBPs) with Peng. et al. reported that circGALNT16 binds to the KH3 domain of hnRNPK to promote SUMOylation of hnRNPK and subsequently restrains colorectal cancer progression [36]. Additionally, Yang. et al. demonstrated that circ-HuR interacts with CCHC-type zinc finger nucleic acid binding protein (CNBP) and results in decreased transcription of HuR in gastric cancer [37]. To better illustrate the mechanism of circSTX6, we performed RNA pull-down and mass spectrometry assays. Combined with the results of silver staining experiments, we verified an RBP of circSTX6 named CUL2. Then, we performed co-IP and ubiquitination assays, which demonstrated that circSTX6 competitively binds with CUL2 and inhibits VHL-EloBC-CUL2-Rbx1 complex-dependent ubiquitination of HIF1A.

HIF1A is a significant regulator of various malignancies by regulating numerous genes involved in glycolysis, cell proliferation and metastasis in the hypoxic microenvironment. However, little is known about its biological functions in normoxic conditions. HIF1A was reported to be regulated by the VHL complex in the existence of oxygen [38–40], and our study also revealed that HIF1A was involved in the regulation of MYH9 transcription in PDAC in normoxic conditions. Data from GEPIA and qRT–PCR results from tissues and cells from our center showed a positive correlation between HIF1A and MYH9. Combining the predictive results of JASPAR, we hypothesized that HIF1A may participate in the transcription of MYH9. The subsequent dual-luciferase and CHIP assays verified this hypothesis. Mechanistically, we confirmed that circSTX6 combined with CUL2 to inhibit VHL-EloBC-CUL2-Rbx1 complex-dependent ubiquitination of HIF1A and subsequently regulated the transcription of MYH9.

## Conclusions

In summary, for the first time, we identified a novel circRNA, circSTX6, which may be a potentially predictive biomarker for PDAC. Our results demonstrate that circSTX6 participates in the posttranscriptional and transcriptional regulation of MYH9 by sponging miR-449b-5p and inhibiting VHL-EloBC-CUL2-Rbx1 complex-dependent ubiquitination of HIF1A, respectively, and subsequently promotes the proliferation and metastasis of PDAC (Fig. 9k). Collectively, our study explores the oncogenic role of circSTX6 in PDAC, and the results suggest that it could serve as a potential biomarker and therapeutic target for the management of PDAC.

## Supplementary Information

**Additional file 1: Table S1.** The RNA-seq results of all differentially expressed circRNAs in five pairs of T4 patients with different survival time.

**Additional file 2: Table S2.** The RNA-seq results of all differentially expressed miRNAs in five pairs of T4 patients with different survival time.

**Additional file 3: Table S3.** The RNA-seq results of all differentially expressed mRNAs in five pairs of T4 patients with different survival time.

**Additional file 4: Table S4.** Primers, probes and siRNAs used in the present study.

**Additional file 5: Table S5.** Antibodies used in the present study.

**Additional file 6: Table S6.** List of possible circSTX6-interacting proteins identified by mass spectrometry.

**Additional file 7: Fig. S1.** CircSTX6 promotes the proliferation, migration and invasion of PDAC cells in vitro. a. The expression levels of circSTX6 and STX6 mRNA in HPNE and PDAC cell lines. β-actin served as the internal control. b-c. CCK-8 assays of CFPAC-1 cells were performed to evaluate cell proliferation. d-e. EdU assays of CFPAC-1 cells were performed to evaluate cell proliferation. Original magnification 400 ×. Scale bar = 50 μm. f-g. Transwell assays in circSTX6 knockdown and circSTX6-overexpressing CFPAC-1 cells. Original magnification 200 ×. Scale bar = 100 μm. h-i. Wound healing assays were used to investigate the migratory abilities of circSTX6-overexpressing and circSTX6 knockdown CFPAC-1 cells. Original magnification 200 ×. Scale bar = 100 μm. j-k. Positive controls for FISH assays. 18 S for the cytoplasm and U6 for the nucleus. Original magnification 400 ×. Scale bar = 50 μm. (Values are expressed as the means ± SDs; *P < 0.05, **P < 0.01 and ***P < 0.001)

**Additional file 8: Fig. S2.** The oncogenic effects of circSTX6 were reversed by miR-449b-5p. a. Transwell assays revealed that the migratory and invasive abilities promoted by circSTX6 overexpression in PANC-1 cells were reversed by miR-449b-5p mimics. b. Transwell assays revealed that the migratory and invasive abilities suppressed by circSTX6 knockdown in CFPAC-1 cells were reversed by the miR-449b-5p inhibitor. c. MiR-449b-5p mimics reversed the promotive effects of circSTX6 overexpression in EdU assays. d. The miR-449b-5p inhibitor reversed the suppressive effects of circSTX6 knockdown in EdU assays. e. Colony formation assays revealed that the effects of circSTX6 in PDAC cells could be reversed by the transfection of miR-449b-5p mimics or inhibitor. (a-b, original magnification 200 ×. Scale bar = 100 μm. c-d, original magnification 400 ×. Scale bar = 50 μm. Values are expressed as the means ± SDs; *P < 0.05, **P < 0.01 and ***P < 0.001)

**Additional file 9: Fig. S3.** HIF1A is indirectly regulated by circSTX6. a-d. The results of transwell and wound healing assays revealed that HIF1A knockdown suppressed and HIF1A overexpression induced the migratory and invasive abilities of PDAC cells, and these effects could be reversed by the transfection of circSTX6 siRNA or plasmid. e-i. Colony formation and CCK-8 assays showed that HIF1A knockdown suppressed and HIF1A overexpression induced the proliferative abilities of PDAC cells, and these effects could be reversed by the transfection of circSTX6 siRNA or plasmid. (**a**-**d**, original magnification 200 ×. Scale bar = 100 μm. Values are expressed as the means ± SDs; *P < 0.05, **P < 0.01 and ***P < 0.001.)

## Data Availability

All data generated or analyzed during this study are included in this published article.

## Abbreviations

circRNA: Circular RNA
PDAC: Pancreatic ductal adenocarcinoma
qRT-PCR: Quantitative reverse transcription polymerase chain reaction
RIP: RNA immunoprecipitation
FISH: Fluorescent in situ hybridization
miRNA: microRNA
CUL2: Cullin 2
MYH9: non-muscle myosin heavy chain 9
HIF1A: Hypoxia-inducible factor 1-alpha
RBPs: RNA binding proteins
HPNE: Normal human pancreatic ductal cell line
AGO2: Argonaute-2
3’UTR: 3′-untranslated regions
COL12A1: Collagen alpha-1(XII) chain
CHIP: Chromatin immunoprecipitation
CCK-8: Cell count kit-8
EdU: 5-ethynyl-20-deoxyuridine
PBS: Phosphate buffered saline
RISC: RNA-induced silencing complex
IHC: Immunohistochemistry
HE: Hematoxylin and eosin
VHL: Von Hippel-Lindau
EloB: Elongin B
EloC: Elongin C
Rbx1: E3 ubiquitin-protein ligase RBX1
CNBP: CCHC-type zinc finger nucleic acid binding protein
