# Supplementary figures and images for: CircSTX6 promotes pancreatic ductal adenocarcinoma progression by sponging miR-449b-5p and interacting with CUL2
Source: Mol Cancer. 2022 Jun 1;21:121. doi: 10.1186/s12943-022-01599-5 (PMC9158112; doi:10.1186/s12943-022-01599-5)

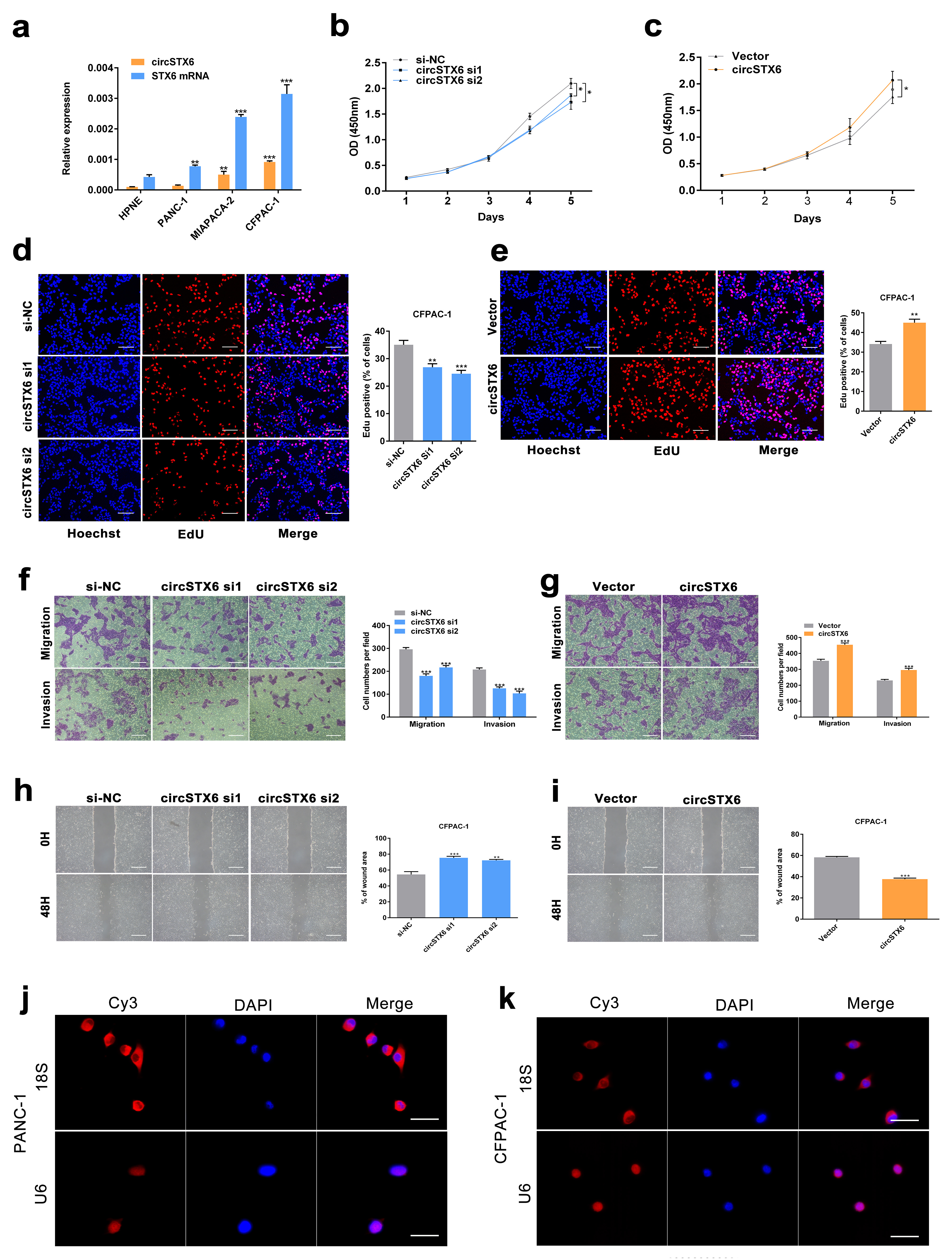

Supplement: Supplementary file 7 — Additional file 7: Fig. S1. CircSTX6 promotes the proliferation, migration and invasion of PDAC cells in vitro. a. The expression levels of circSTX6 and STX6 mRNA in HPNE and PDAC cell lines. β-actin served as the internal control. b-c. CCK-8 assays of CFPAC-1 cells were performed to evaluate cell proliferation. d-e. EdU assays of CFPAC-1 cells were performed to evaluate cell proliferation. Original magnification 400 ×. Scale bar = 50 μm. f-g. Transwell assays in circSTX6 knockdown and circSTX6-overexpressing CFPAC-1 cells. Original magnification 200 ×. Scale bar = 100 μm. h-i. Wound healing assays were used to investigate the migratory abilities of circSTX6-overexpressing and circSTX6 knockdown CFPAC-1 cells. Original magnification 200 ×. Scale bar = 100 μm. j-k. Positive controls for FISH assays. 18 S for the cytoplasm and U6 for the nucleus. Original magnification 400 ×. Scale bar = 50 μm. (Values are expressed as the means ± SDs; *P < 0.05, **P < 0.01 and ***P < 0.001) [file 12943_2022_1599_MOESM7_ESM.jpg]

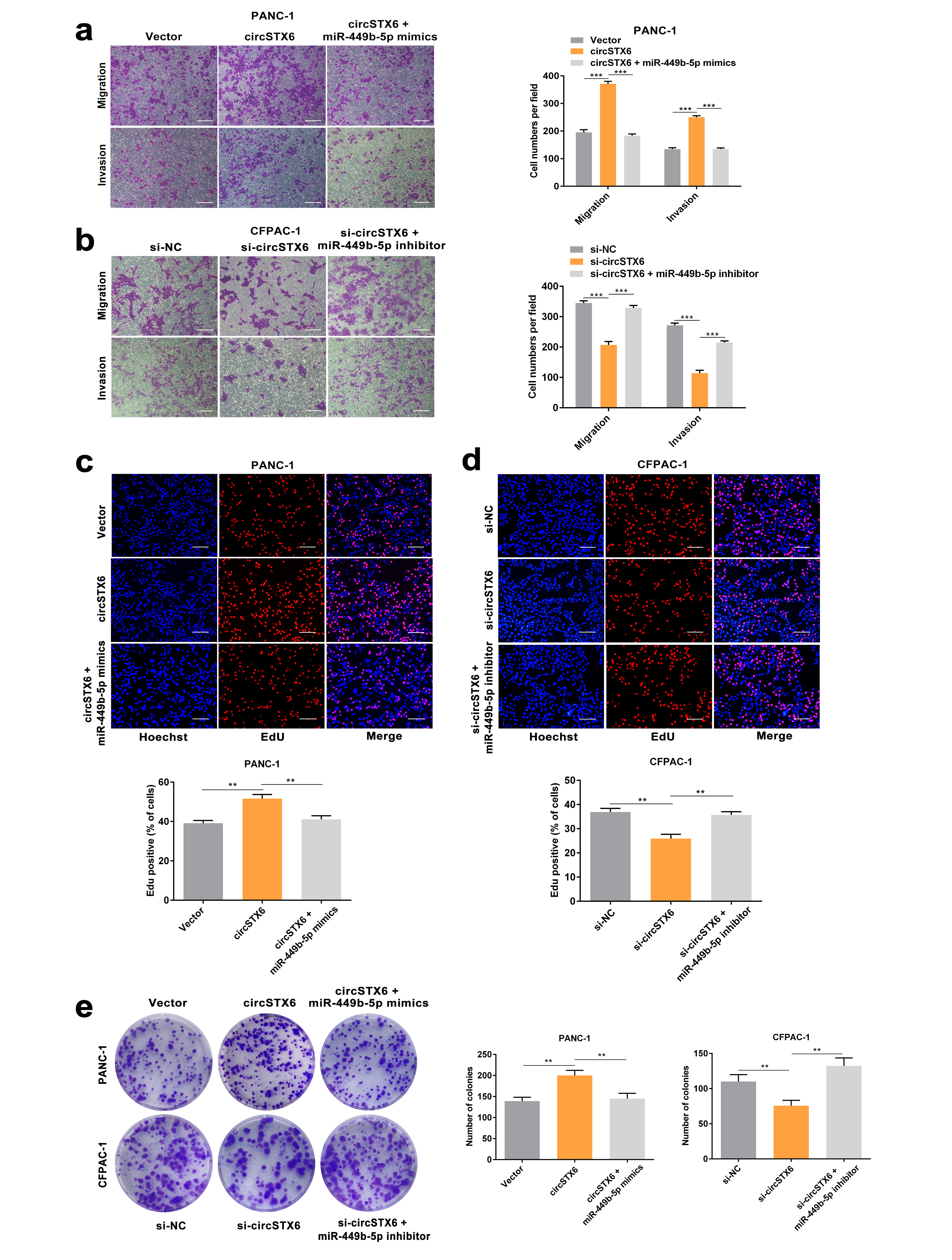

Supplement: Supplementary file 8 — Additional file 8: Fig. S2. The oncogenic effects of circSTX6 were reversed by miR-449b-5p. a. Transwell assays revealed that the migratory and invasive abilities promoted by circSTX6 overexpression in PANC-1 cells were reversed by miR-449b-5p mimics. b. Transwell assays revealed that the migratory and invasive abilities suppressed by circSTX6 knockdown in CFPAC-1 cells were reversed by the miR-449b-5p inhibitor. c. MiR-449b-5p mimics reversed the promotive effects of circSTX6 overexpression in EdU assays. d. The miR-449b-5p inhibitor reversed the suppressive effects of circSTX6 knockdown in EdU assays. e. Colony formation assays revealed that the effects of circSTX6 in PDAC cells could be reversed by the transfection of miR-449b-5p mimics or inhibitor. (a-b, original magnification 200 ×. Scale bar = 100 μm. c-d, original magnification 400 ×. Scale bar = 50 μm. Values are expressed as the means ± SDs; *P < 0.05, **P < 0.01 and ***P < 0.001) [file 12943_2022_1599_MOESM8_ESM.jpg]

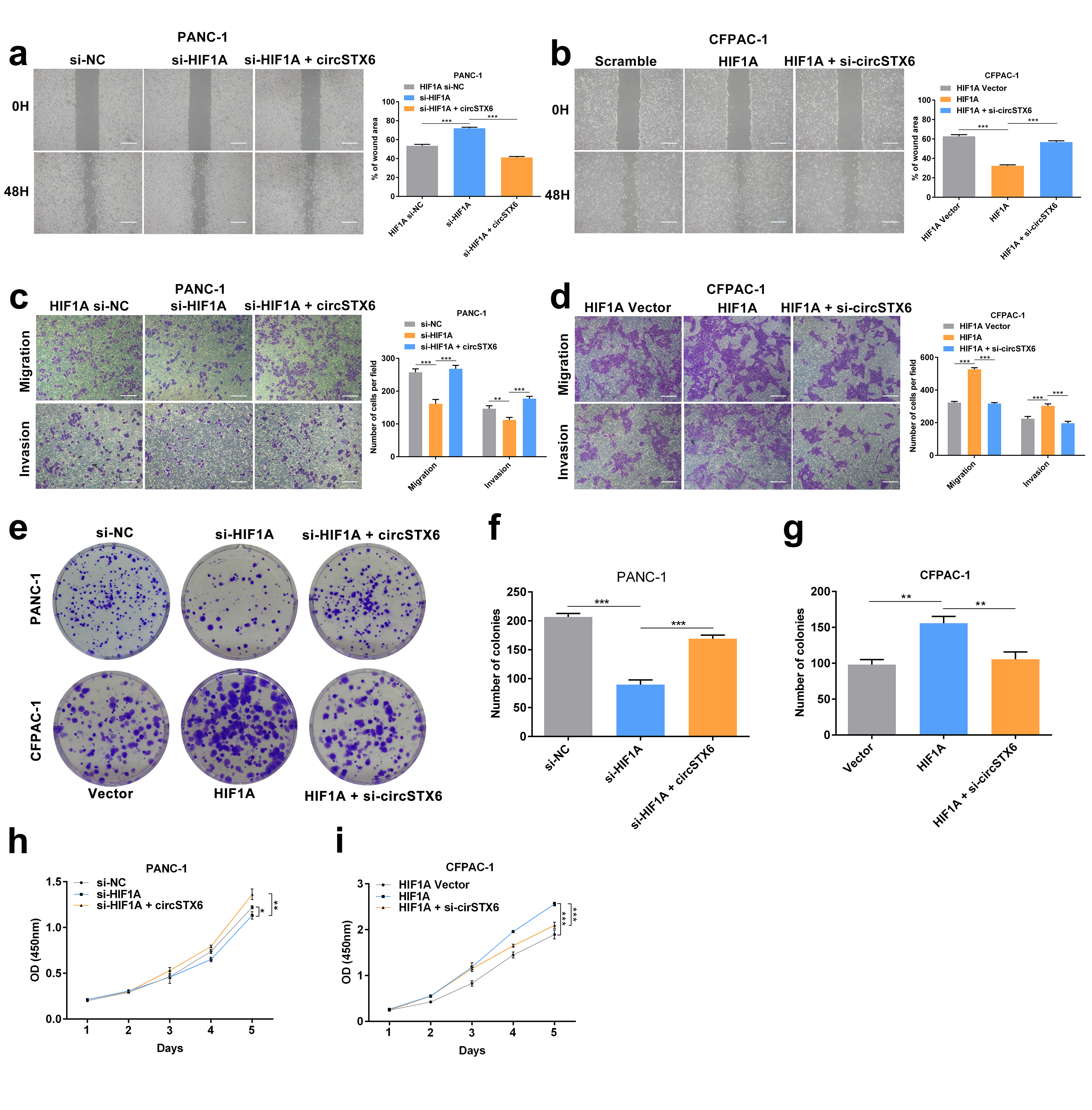

Supplement: Supplementary file 9 — Additional file 9: Fig. S3. HIF1A is indirectly regulated by circSTX6. a-d. The results of transwell and wound healing assays revealed that HIF1A knockdown suppressed and HIF1A overexpression induced the migratory and invasive abilities of PDAC cells, and these effects could be reversed by the transfection of circSTX6 siRNA or plasmid. e-i. Colony formation and CCK-8 assays showed that HIF1A knockdown suppressed and HIF1A overexpression induced the proliferative abilities of PDAC cells, and these effects could be reversed by the transfection of circSTX6 siRNA or plasmid. (a-d, original magnification 200 ×. Scale bar = 100 μm. Values are expressed as the means ± SDs; *P < 0.05, **P < 0.01 and ***P < 0.001.) [file 12943_2022_1599_MOESM9_ESM.jpg]
